# Early Deletion of *Neurod1* Alters Neuronal Lineage Potential and Diminishes Neurogenesis in the Inner Ear

**DOI:** 10.3389/fcell.2022.845461

**Published:** 2022-02-17

**Authors:** Iva Filova, Romana Bohuslavova, Mitra Tavakoli, Ebenezer N. Yamoah, Bernd Fritzsch, Gabriela Pavlinkova

**Affiliations:** ^1^ Laboratory of Molecular Pathogenesis, Institute of Biotechnology CAS, Vestec, Czechia; ^2^ Department of Physiology and Cell Biology, Institute for Neuroscience, University of Nevada, Reno, NV, United States; ^3^ Department of Biology, University of Iowa, Iowa City, IA, United States

**Keywords:** Foxg1, cochlear nuclei, Neurod1, vestibular system, auditory system, neurons, hair cells, projections

## Abstract

Neuronal development in the inner ear is initiated by expression of the proneural basic Helix-Loop-Helix (bHLH) transcription factor *Neurogenin1* that specifies neuronal precursors in the otocyst. The initial specification of the neuroblasts within the otic epithelium is followed by the expression of an additional bHLH factor, *Neurod1.* Although NEUROD1 is essential for inner ear neuronal development, the different aspects of the temporal and spatial requirements of NEUROD1 for the inner ear and, mainly, for auditory neuron development are not fully understood. In this study, using Foxg1^Cre^ for the early elimination of *Neurod1* in the mouse otocyst, we showed that *Neurod1* deletion results in a massive reduction of differentiating neurons in the otic ganglion at E10.5, and in the diminished vestibular and rudimental spiral ganglia at E13.5. Attenuated neuronal development was associated with reduced and disorganized sensory epithelia, formation of ectopic hair cells, and the shortened cochlea in the inner ear. Central projections of inner ear neurons with conditional *Neurod1* deletion are reduced, unsegregated, disorganized, and interconnecting the vestibular and auditory systems. In line with decreased afferent input from auditory neurons, the volume of cochlear nuclei was reduced by 60% in *Neurod1* mutant mice. Finally, our data demonstrate that early elimination of *Neurod1* affects the neuronal lineage potential and alters the generation of inner ear neurons and cochlear afferents with a profound effect on the first auditory nuclei, the cochlear nuclei.

## Introduction

The inner ear is a highly organized structure of interlinked channels and chambers built to encode sound, space orientation, and motion (Goodrich, 2016; Elliott et al., 2021). The sensory organs are represented by five vestibular epithelia (the maculae of utricle and saccule, and the cristae of the three semicircular canal ampullae), and, in mammals, by the auditory sensory organ of the cochlea, the organ of Corti (Rubel and Fritzsch, 2002; Fritzsch et al., 2013). The sensory receptors for hearing and balance are mechanotransducing hair cells (Elliott et al., 2018). Apart from sensory hair cells, the sensory epithelium consists of non-sensory supporting cells necessary for hair cells’ development, function, and maintenance. The sensory epithelia are innervated by neurons of the inner ear of two different ganglia, the vestibular and spiral ganglia. The vestibular ganglion is located in the lateral portion of the internal auditory meatus, with the superior division innervating the utricle, the superior semi-circular canal, and the lateral semi-circular canal, while the inferior region of the vestibular ganglion innervates the saccule and the posterior semi-circular canal (Khan and Chang, 2013). The auditory spiral ganglion twists along the length of the cochlear duct with peripheral neuronal processes innervating hair cells within the auditory sensory epithelium and central axons merging into the cochlear nerve (Pavlinkova, 2020). These inner ear neurons transmit received information from the sensory cells to the central nervous system.

All sensory organs of the inner ear and its associated sensory ganglia derive from a single embryonic source, the otic placode. Neurons seem to be the first differentiated cells in the developing inner ear in all species examined (Fritzsch and Straka, 2014). The initial specification of the neuroblasts within the otic epithelium is followed by the delamination of neuroblasts as early as embryonic day nine (E9) in the mouse embryo. The neuronal cells form the inner ear ganglion, later separating into the vestibular and spiral ganglia between E9.5 and E14.5 (Ma et al., 2000). Cells fated to develop as sensory hair cells and supporting cells of the sensory epithelia arise from the prosensory domain of the otocyst (Wu and Kelley, 2012). Deciphering the developmental mechanisms of these specialized neurosensory cells of the inner ear is a central focus of cell-based therapy strategies.

The development of sensory hair cells, neurons, and non-sensory cells in the inner ear is regulated by a network of signaling pathways and transcription factors. Proneural atonal-related basic helix-loop-helix (bHLH) transcription factors, ATOH1, NEUROGENIN1 (NEUROG1), and NEUROD1, are critical players in inner ear development. ATOH1 is essential for sensory cell differentiation (Bermingham et al., 1999). NEUROG1*,* the first bHLH factor upregulated in the otocyst, initiates the specification and differentiation of inner ear neurons (Ma et al., 2000). *Neurog1* null mice lack inner ear neurons and have a massive loss of sensory epithelia through the alteration of *Atoh1* expression (Ma et al., 2000; Matei et al., 2005; Kim et al., 2001).

Additionally, NEUROG1 activates the expression of the downstream bHLH gene, *Neurod1*, which is essential for neuronal differentiation (Kim et al., 2001). *Neurod1* null mice exhibit severely impaired differentiation of auditory and vestibular neurons (Liu et al., 2000a; Kim et al., 2001) but they also suffer from other neuronal developmental defects (Miyata et al., 1999; Liu et al., 2000b) and a severe diabetic phenotype (Kim et al., 2001). Therefore, conditional *Neurod1* deletion mutants were generated to investigate more specifically its role in inner ear neuronal development and hearing function. Conditional *Pax2* ^*Cre*^ *; Neurod1* ^*f/f*^ deletion mice showed abnormalities in the formation of inner ear ganglia, disorganized cochlear innervation, and unsegregated vestibular and spiral ganglion afferents (Jahan et al., 2010a). Unfortunately, *Pax2* ^*Cre*^ *; Neurod1* ^*f/f*^ mice have *Neurod1* deletion in the ear and the central auditory nuclei, limiting the evaluation of spiral ganglion neuronal viability and central projections and hampering physiological assessment of the wiring defects. Furthermore, Pax2^Cre^ activity decreases with age, starting at E10.5 and producing uneven deletion effects (Duncan and Fritzsch, 2013). A delayed conditional *Neurod1* deletion using *Isl1* ^*Cre*^ eliminates *Neurod1* from differentiating inner ear neurons and retains *Neurod1* expression in the auditory nuclei and midbrain (Macova et al., 2019). The *Isl1* ^*Cre*^ *; Neurod1* ^*f/f*^ mutants form the unsegregated and disorganized peripheral projection map of spiral ganglion neurons with altered sensory information processing in the central auditory pathway (Macova et al., 2019; Filova et al., 2020). However, detailed insights into the effects of an early absence of *Neurod1* in otic neuroblasts are lacking.

To go beyond existing data and reveal the dependency of early neuronal development on the otocyst-expressed NEUROD1 protein*,* we chose the *Foxg1* ^*Cre*^ transgene to eliminate *Neurod1. Foxg1* ^*Cre*^ has been demonstrated to lead to earlier and more profound recombination than other Cre drivers expressed in the ear placode and the developing ear (Duncan and Fritzsch, 2013; Dvorakova et al., 2020). *Foxg1* ^*Cre*^ is not expressed in the auditory and vestibular nuclei to possibly affect neuronal viability in the inner ear (Hébert and McConnell, 2000; Bérubé et al., 2005; Kasberg et al., 2013; Abrams and Reiter, 2021). In this study, we revisited the embryonic phenotype of *Neurod1* deletion mice to determine NEUROD1 requirements for the generation and survival of neuroblasts and, overall, for early inner ear development.

## Material and Methods

### Experimental Animals

All experiments with mice were approved by the Animal Care and Use Committee of the Institute of Molecular Genetics, Czech Academy of Sciences. Animals were housed in a controlled environment with 12-h light/dark cycles and free access to water and food. For obtaining the mouse model with conditional deletion of *Neurod1*, we crossbred Foxg1^Cre/+^ knock-in/knock-out mice (129(Cg)-Foxg1^tm1(cre)Skm^/J; #004337 The Jackson Laboratory) with mice carrying the *Neurod1* gene flanked by loxP sites (*Neurod1* ^*loxP/loxP*^ (Goebbels et al., 2005)). Mice were genotyped using DNA isolated from the tail. The following PCR primers were used: Cre Forward 5′-GCC TGC ATT ACC GGT CGA TGC AAC GA-3′ and Cre Reverse 5′-GTG GCA GAT GGC GCG GCA ACA CCA TT-3′ with a 700 bp product; Neurod1 Forward 5′-ACC ATG CAC TCT GTA CGC ATT-3′ and Neurod1 Reverse 5′-GAG AAC TGA GAC ACT CAT CTG-3′ with a 400 bp product for the WT allele or 600 bp for the allele containing loxP sites. The noon of the day the vaginal plug was found was defined as embryonic day 0.5 (E0.5). Mice of both sexes were used for experiments.

### Morphological Evaluation of the Inner Ear, Cochlear Nucleus, and Inferior Colliculus

Pregnant mice were sacrificed at noon, and embryos or dissected embryonic tissues were then fixed in 4% paraformaldehyde (PFA) in phosphate-buffered saline (PBS). Neonates were transcardially perfused with PBS followed by 4% PFA. Brains and inner ear were dissected from the skull and fixed in 4% PFA. For longtime storage, tissues were kept in methanol at −20°C. Embryos or tissues were stained as whole mounts or 80-μm vibratome sections (Leica VT1000 S). Samples were blocked in a blocking solution consisting of 2.5% donkey or goat serum, 0.5% Tween20, and 0.1% Triton X-100 for at least 1 h at room temperature. Next, samples were incubated with primary antibodies (Supplementary Table S1) diluted in blocking solution for 72 h at 4°C. After several washes with PBS, samples were incubated with secondary antibodies (Supplementary Table S1) for 24 h at 4°C. Finally, cell nuclei were labeled with Hoechst 33258 (Merck 861405; always showed as blue staining) diluted 1:2000 in PBS. After staining, samples were mounted in Aqua-Poly/Mount (Polysciences 18606) or prepared an anti-fade medium. Images were taken on Zeiss LSM 880 NLO inverted confocal microscope and Nikon CSU-W1 spinning disk confocal microscope. NIS-Elements, ImageJ, and ZEN software were used for image processing.

The area of the inner ear ganglion at E10.5 was determined as ISL1^+^ area in 80-μm vibratome transverse sections of the embryo (dotted area). For quantification, sections with the two largest areas containing the inner ear ganglion were picked for each embryo, and outliers were excluded (*n* = 10 embryos/genotype; 16 sections/genotype). In the area of inner ear ganglion, ISL1^+^ cells were counted in the same sections using the Cell Counter ImageJ plugin. For quantifying proliferation and apoptosis, anti-Phospho-Histone H3 and Cleaved Caspase3 antibodies were used, respectively. Phospho-Histone H3 or Cleaved Caspase3 positive cells were counted in the ISL1^+^ ganglion area in vibratome sections at E10.5. For each genotype two sections per embryo were selected (*n* = 5 embryos/genotype). We compared the size of E18.5 auditory and vestibular organs between controls and mutants. Inner ear whole mounts were stained with an antibody against hair cell marker, MyosinVIIa. The cochlear length was established as a line between inner and outer hair cells from the apical tip to the base. The length of the organ of Corti was measured using the “Measure line” ImageJ plugin. The size of the vestibular organ epithelium was measured using immunolabeling of the MyosinVIIa^+^ area using “Polygon selection,” and “Measure” functions of ImageJ. Volumes of the cochlear nucleus (CN) and inferior colliculus (IC) were determined by using 80-μm coronal brain sections (*n* = 5 brains/genotype). The areas of the left and right CNs and ICs were measured in all sections, and the total volume of the brain structures was calculated. Parvalbumin^+^ bushy cells were counted in 80-μm vibratome sections (image view 134.95 × 134.95 μm) of adult VCN (two sections/one animal and four animals/genotype). Number of parvalbumin^+^ bushy cells per measured area of VCN was determined. To quantify endbulbs of Held, we used vibratome sections of the adult VCN. Three cells with the visually largest VGLUT1^+^ area were selected and measured in each section (two sections/one animal and four animals/genotype). The VGLUT1^+^ area of the synapse was determined per the bushy cell area.

### Lipophilic Dye Tracing

Heads of P0 pups were fixed in 4% PFA in 0.1 M phosphate buffer with 300 mM sucrose (to minimize neuronal swelling) for 48 h at 4°C and processed for dye tracing. To trace projections of the ear to the brain, we used NeuroVue dyes (NV Jade, NVJ; NV Red, NVR) as previously described (Fritzsch et al., 2016; Schmidt and Fritzsch, 2019). Lipophilic dyes were applied using dye*-*soaked filter strips, which provide a more precise and reliable dye application than crystals or dye-injections (Schmidt and Fritzsch, 2019). For tracing inner ear central projections to the brain, the dye was placed into the inner ear to label the spiral ganglion, organ of Corti, and vestibular neurons. The dye was allowed to diffuse for 24–72 h, depending on the embryonic age and dye. Mutant and control samples were always processed in parallel, with an identical dye placement and dye diffusion time. Brains and ears were subsequently microdissected, mounted in glycerol, and imaged using a Leica SP8 confocal microscope. After imaging, some brains and ears were post-fixed in 4% PFA and either processed for immunohistochemistry (*see* above) or sectioned at 100-μm, mounted in glycerol, and imaged to reveal the distribution of projections in sections.

### Scanning Electron Microscopy

Inner ears devoid of cartilage were fixed in 2.5% glutaraldehyde and 2% formaldehyde in 1×PHEM buffer at °C overnight. After that, tissues were washed with 1×PHEM buffer a few times, dehydrated in graded ethanol series, and finally transferred into 100% acetone and dried to a critical point in Leica CPD300 with CO_2_. The dried samples were mounted on regular SEM stubs using conductive carbon and coated with 7 nm of platinum in Leica ACE600. The images were taken using a dual-beam system FEI Helios NanoLab 660 G3 UC scanning electron microscope at 2 kV and 0.2 nA with an Everhart-Thornley secondary electron detector.

### Experimental Design and Statistical Analysis

All comparisons were made between animals with the same genetic background, typically littermates, and we used male and female mice. The number of samples (*n*) for each comparison can be found in the individual method descriptions and in the corresponding figures. Phenotyping and data analysis was performed blind to the genotype of the mice. All values are presented either as the mean ± standard deviation (SD) or standard error of the mean (SEM). For statistical analysis, GraphPad Prism software was used. The differences in the mean were compared using unpaired two-tailed *t*-test or one-way ANOVA tests for statistical evaluation. Significance was determined as *p* < 0.05 (*), *p* < 0.01 (**), *p* < 0.001 (***) or *p* < 0.0001 (****). Complete results of the statistical analyses are included in the figure legends.

## Results

### *Neurod1* Mutants Have Reduced Body Size and Vestibular Dysfunction

*Neurod1* was eliminated by crossing *Neurod1* ^*loxP/loxP*^ mice (Goebbels et al., 2005) with *Foxg1* ^*Cre*/+^ line (Hébert and McConnell, 2000), which has efficient Cre activity during early ear development, starting in the otic placode (Dvorakova et al., 2020). We confirmed *Foxg1* ^*Cre*^ expression in sensory cells and neurons in the inner ear using *tdTomatoAi14* reporter mice (Supplementary Figures S1A,B). Foxg1^Cre^ activity was undetectable in the cochlear nucleus, showing no tdTomato expression in neurons of the cochlear nucleus (Supplementary Figures S1C,D). Homozygous mutant embryos (*Neurod1CKO*; genotype *Foxg1* ^*Cre/+*^ *, Neurod1* ^*loxP/loxP*^) were recovered at the expected Mendelian ratios at all embryonic days examined *in utero* (Supplementary Figure S2A), suggesting no effect of *Neurod1* elimination on embryonic survival. However, postnatal development of homozygous *Neurod1CKO* and heterozygous (*Foxg1* ^*Cre/+*^ *, Neurod1* ^*loxP/+*^) mutants was reduced (Supplementary Figure S2B), with fewer mice identified at the time of genotyping (3 weeks of age) compared to control mice (without Cre allele, genotypes: *Foxg1* ^*+/+*^ *, Neurod1* ^*loxP/+*^ or *Foxg1* ^*+/+*^ *, Neurod1* ^*loxP/loxP*^). *Neurod1CKO* mice were born significantly smaller than their littermates (Supplementary Figure S2C), indicating abnormalities during embryonic development. *Neurod1CKO* failed to thrive from birth to weaning with significant deficits in body size and body weight compared to controls at postnatal day P21. *Neurod1CKO* mice suffered from severe ataxia (Supplementary Movie), and due to rapid deterioration, had to be sacrificed 3–4 weeks after birth. Surprisingly, the decreased gene dose of *Neurod1* in heterozygous mutant mice negatively affected their postnatal development. Although these mice did not present any signs of ataxia, they were significantly smaller than controls.

**FIGURE 1:**
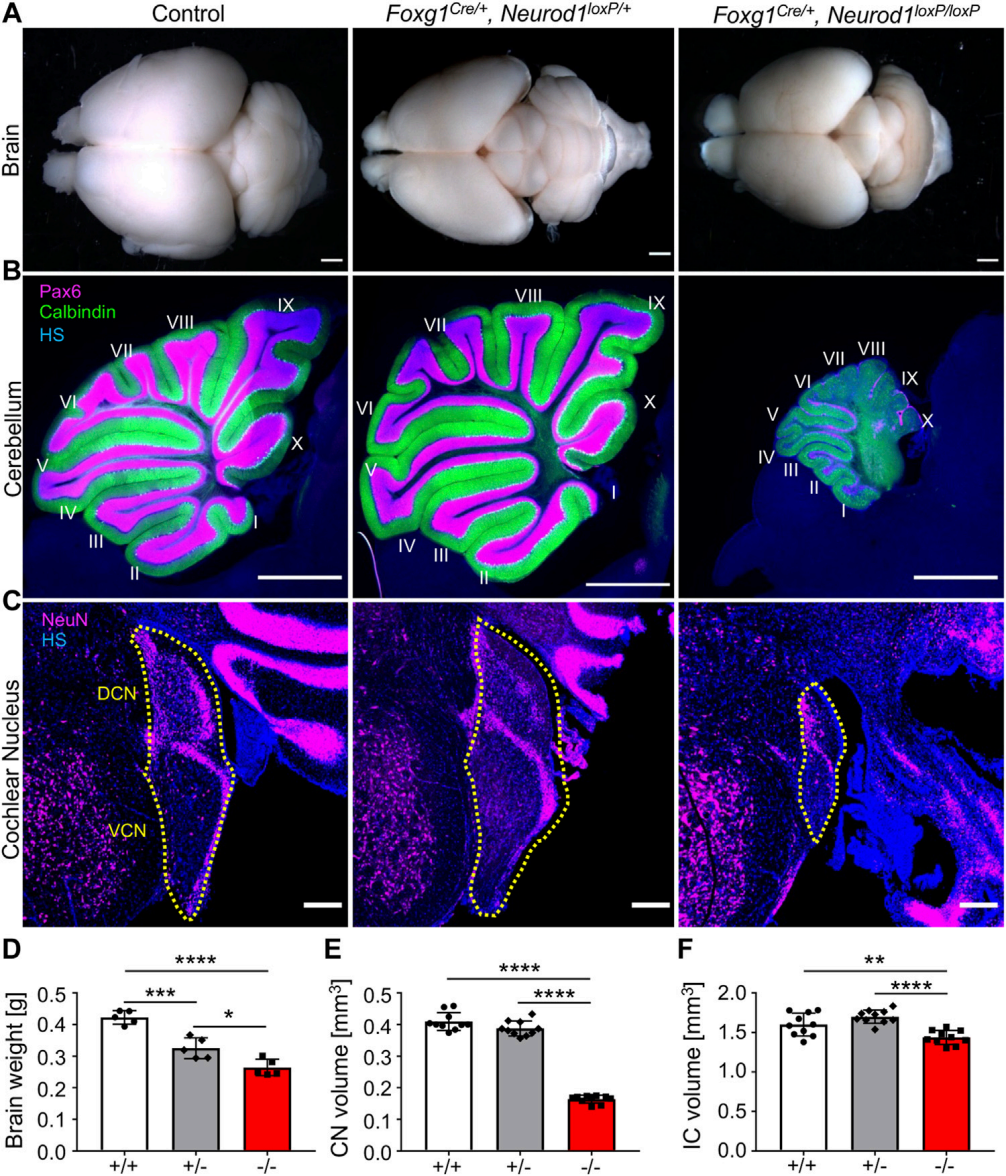
Brain abnormalities are detected in *Neurod1CKO* mutants. **(A**,**D)** Adult brain size was reduced in *Foxg1* ^*Cre/+*^ *, Neurod1* ^*loxP/+*^ heterozygotes (+/−) and *Foxg1* ^*Cre/+*^ *, Neurod1* ^*loxP/loxP*^ homozygous (−/−) mutants compared to controls. **(B)** The *Neurod1CKO* cerebellum was smaller with many Purkinje cells (Calbindin^+^) but with fewer granule cells (Pax6^+^). **(C**,**E**,**F)** The cochlear nucleus’s size and inferior colliculus were significantly reduced in *Neurod1CKO* mutants compared to controls and heterozygotes. The dotted line indicates the boundaries of the cochlear nucleus. The values represent means ± SD, one-way ANOVA, *p ≤ 0.05, **p ≤ 0.01, ***p ≤ 0.001, ****p ≤ 0.0001. Scale bars: 1,000 µm **(A**,**B)**; 200 μm **(C)**. CN, cochlear nucleus; DCN, dorsal cochlear nucleus; HS, Hoechst nuclear staining; IC, inferior colliculus; VCN, ventral cochlear nucleus.

To investigate the reduced postnatal survival of homozygous and heterozygous *Neurod1* mutants, we first evaluated changes in the brain since *Foxg1* ^*Cre*^ is highly expressed in the forebrain. Correspondingly, the size of the forebrain was visibly more petite, and the weight of the brain was significantly reduced in both homozygous and heterozygous *Neurod1* mutants compared to controls (Figures 1A,D). The size and morphology of the cerebellum and the size of the cochlear nucleus were exclusively affected only in *Neurod1CKO* mutants (Figures 1B,C,E,F). Analysis of the gross external morphology of born pups showed no abnormalities in an eye formation, in a shape of the forehead or the snout (Supplementary Figure S2D). *Foxg1* ^*C*re^ is expressed in the olfactory system and eye (Dvorakova et al., 2020), but the aspects of the olfactory epithelium and retina of the visual system were comparable between control and *Neurod1CKO* (Supplementary Figure S3). Our results confirmed the previous study’s conclusions that NEUROD1 is not required for eye (Pennesi et al., 2003) and olfactory embryonic development (Packard et al., 2011).

### Reduced Sensory Epithelia and a Severe Loss of Spiral Ganglion Neurons are Found in the *Neurod1CKO* Inner Ear

**FIGURE 2:**
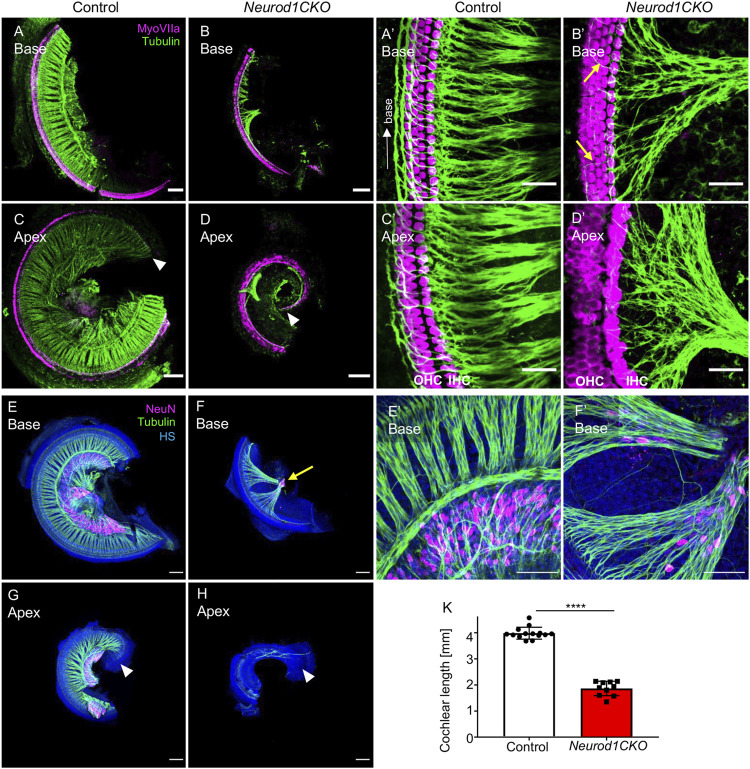
The elimination of *Neurod1* results in the rudimental spiral ganglion, disorganized sensory epithelium, and the shortened cochlea. **(A**–**D)** Representative images of whole-mount immunolabeling of the cochlear base and apex with anti-MyosinVIIa (MyoVIIa, a marker of hair cells) and anti-α-tubulin (nerve fibers) at E18.5. Arrowheads indicate the apical end. **(A’**–**D’)** Higher-magnification images show a dense network of radial fibers, three rows of outer hair cells (OHC), and one row of inner hair cells (IHC), forming the organ of Corti in the control cochlea. Note the formation of parallel outer spiral bundles turned toward the base in the control cochlea (arrow in **A’**). In *Neurod1CKO*, radial fibers are severely reduced and disorganized, turning randomly towards the base or apex (arrows in **B’**). The increased number of OHC rows in the apex indicates the disorganized sensory epithelium of the *Neurod1CKO* cochlea (**D’**). **(E**–**H)** The cochlea’s whole-mounted basal and apical half immunolabeled with anti-NeuN (a nuclear marker of differentiated neurons) and anti-tubulin (nerve fibers) shows NeuN^+^ neurons forming spiral ganglion in control at P0. In contrast, only a small cluster of NeuN^+^ neurons is found in the *Neurod1CKO* cochlea (arrow in **F**). Note the reduced size of the cochlea and massive reduction of innervation. **(E’**, **F’)** Higher-magnification images of the base show the aberrant distribution of NeuN^+^ neurons entangled with radial fibers in *Neurod1CKO* compared to the spiral ganglion neurons restricted to the Rosenthal’s canal in the control cochlea. **(K)** The length of the organ of Corti is significantly shorter in *Neurod1CKO* compared to control littermates. Error bars represent mean ± SD; unpaired *t-*test, ****p ≤ 0.0001 (*n* ≥ 5/genotype). Scale bars: 100 μm **(A**–**H)**, 25 μm **(A**’–**D’)**, 50 μm **(E’**,**F’)**. HS, Hoechst nuclear staining.

Next, we evaluated the inner ear morphology of heterozygous and homozygous *Neurod1* mutants. Radial fibers, spiral ganglion, and the organ of Corti of heterozygous *Foxg1* ^*Cre/+*^ *, Neurod1* ^*loxP/+*^ mice were comparable to the morphology of the control cochlea (Supplementary Figure S4). The formation and size of sensory epithelia of the vestibular-end organs and innervation of Foxg*1* ^*Cre/+*^ *, Neurod1* ^*loxP/+*^ were also similar to control mice. Thus, the normal inner ear morphology and the normal vestibular behavior indicate that eliminating one *Neurod1* allele did not affect the inner ear development of the heterozygous *Neurod1* mutant. In contrast, the cochlea of *Neurod1CKO* was severely shortened, with the length of the organ of Corti reaching only 47% of the littermate control at E18.5 (Figures 2A–D,K). Besides the length of the cochlea, the most noticeable deficiency in *Neurod1CKO* was a reduction of radial fibers. Higher-magnification images showed substantially decreased and disorganized innervation of the sensory epithelium with a few fibers of outer spiral bundles turning randomly toward the base or apex (Figure 2B’) compared to dense parallel outer spiral bundles directed toward the base in the control cochlea (Figure 2A’). Additionally, abnormalities in the apical epithelium of *Neurod1CKO* with multiple hair cell rows were noticeable compared to the control cochlea (Figures 2C,D). Correspondingly, all sensory epithelia of the vestibular end-organs of *Neurod1CKO* were significantly reduced compared to controls (Supplementary Figure S5).

In line with severe reduction of innervation in the *Neurod1CKO* cochlea, we found only a rudiment of the spiral ganglion at P0, represented by a clump of NeuN^+^ neurons (Figures 2F,H), compared to the spiral ganglion neurons located in the Rosenthal’s spiral canal in controls (Figures 2E,G). In contrast to controls, some NeuN^+^ neurons were entangled with fibers in the *Neurod1CKO* cochlea (Figures 2E’,F’). Some of these fibers were type Ia neuron afferents, as shown by anti-calretinin labeling of the cochlea in adult mice (Supplementary Figure S6). Thus, these results indicate a significant loss of neurons, severe abnormalities in the formation of the spiral ganglion and innervation.

### Early Inner Ear Neuronal Development Is Altered in *Neurod1CKO*

**FIGURE 3:**
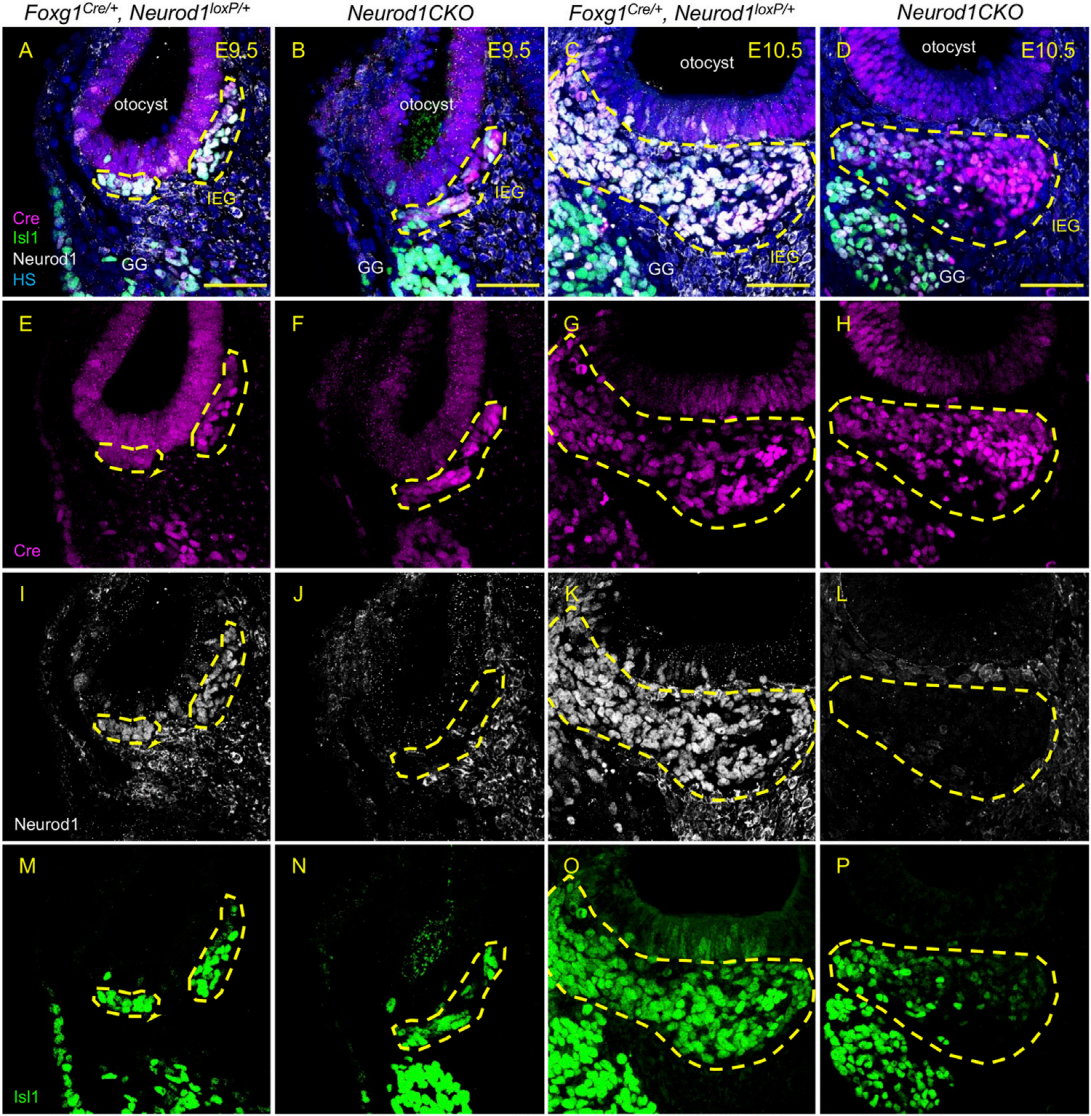
Efficient elimination of *Neurod1* by Foxg1-Cre is shown in delaminating neuroblasts and the inner ear ganglion. Cre recombinase (red) was detected as early as E9.5 in mouse *Foxg1* ^*Cre/+*^ otocyst **(A**,**B**,**E**,**F)**. Neurod1 (white) is eliminated using Foxg1^Cre^ in mutants at E9.5 **(J)** as well as at E10.5 **(L)**, while in controls, NEUROD1 is expressed in delaminating neurons **(I**,**K)** that are co-labeled by Isl1 antibody (green; **M**–**P**). The area of the inner ear ganglion is delineated by the dotted line. Scale bars: 50 µm. GG, geniculate ganglion; HS, Hoechst nuclear staining; IEG, inner ear/otic ganglion.

**FIGURE 4:**
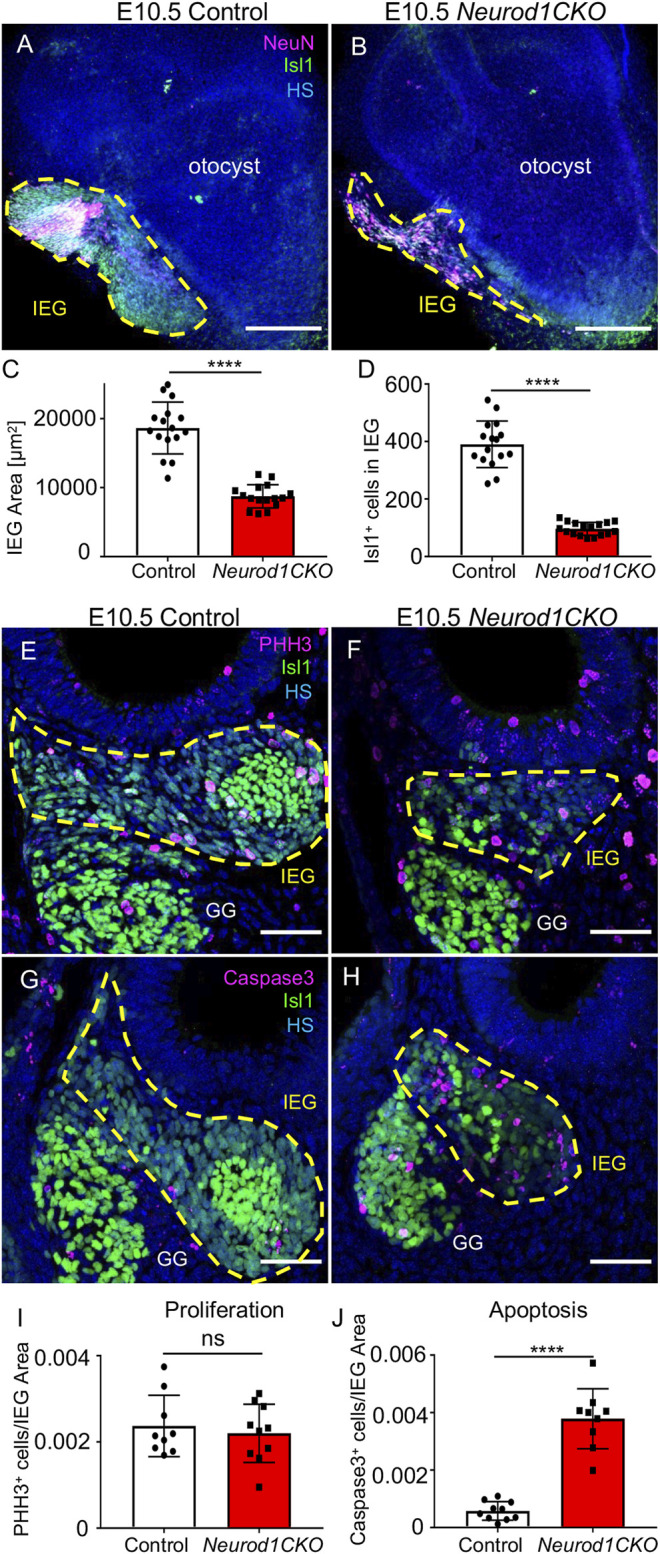
Diminished inner ear ganglion and massive apoptosis are detected in *Neurod1CKO* at E10.5. **(A**,**B)** Representative whole-mount immunolabeling of the otocyst shows the size of otic ganglia (dotted line area) with anti-Isl1 and anti-NeuN (a nuclear marker of neurons). **(C**,**D)** Quantification of the ganglion size and number of ISL1^+^ neurons in the inner ear ganglion. The values represent mean ± SD, *t*-test, ****p ≤ 0.0001 (*n* = 10 embryos/genotype, 16 vibratome sections/embryo). **(E**,**F)** Immunohistochemistry for Phosphohistone H3 (PHH3) and ISL1 shows proliferating neurons in the otic ganglion (dotted line area) in the vibratome sections of *Neurod1CKO* and control embryos. **(G**,**H)** Anti-Cleaved Caspase3-labeled apoptotic cells are shown in the ISL1^+^ ganglion area (delineated by the dotted line) at E10.5. **(I)** A number of proliferating neurons and **(J)** apoptotic neurons per the ganglion area were counted in the vibratome sections of *Neurod1CKO* and control embryos. The values represent means ± SD, *t-*test; ns, not significant; ****p ≤ 0.0001 (*n* = 5 embryos/genotype). Scale bars: 200 µm **(A**,**B)**; 50 µm **(E**–**H)**. GG, geniculate ganglion; HS, Hoechst nuclear staining; IEG, inner ear/otic ganglion.

Having recognized a severe loss of neurons of *Neurod1CKO*, we wanted to determine the effects of *Neurod1* elimination on early inner ear development. First, we determined the efficiency and relative onset of *Neurod1* deletion in the otocyst of *Neurod1CKO*. We could not detect any NEUROD1 expressing cells in the ear epithelium or any NEUROD1^+^ delaminating neuroblasts in *Neurod1CKO* at E9.5 and E10.5 (Figure 3). The elimination of NEUROD1 protein was consistent with the regional expression of Cre recombinase under *Foxg1*. ISL1 expressing neurons were detected in the otic ganglion of *Neurod1CKO.* However, the number of ISL1^+^ cells was noticeably reduced as early as E9.5, indicating a negative effect of *Neurod1* elimination on early neurogenesis in the inner ear. Note the decreased ISL1^+^ neuroblasts in the epithelium of the *Neurod1CKO* otocyst compared to the control. Second, we measured the size of the inner ear ganglion. The *Neurod1CKO* ganglion was reduced by 53% compared to control embryos at E10.5 (Figures 4A–D). The number of neurons expressing ISL1 in *Neurod1CKO* was decreased on average by about 80% (96 ± 5, *n* = 10 embryos) compared to controls (390 ± 20, *n* = 10 embryos). Interestingly, the number of proliferating cells in the ganglion was similar between the control and *Neurod1CKO* inner ear (Figures 4E,F,I). However, we detected massive apoptosis in neurons of the *Neurod1CKO* ganglion. The number of apoptotic cells was six times higher in the mutant than in the control otic ganglion (Figures 4G,H,J). These results indicate that NEUROD1 is important for neurogenesis and neuronal survival in the early stages of inner ear neuronal development.

### Depleted Inner Ear Neurons of *Neurod1CKO* Form an Aberrant Cochlear and Vestibular Ganglia

**FIGURE 5:**
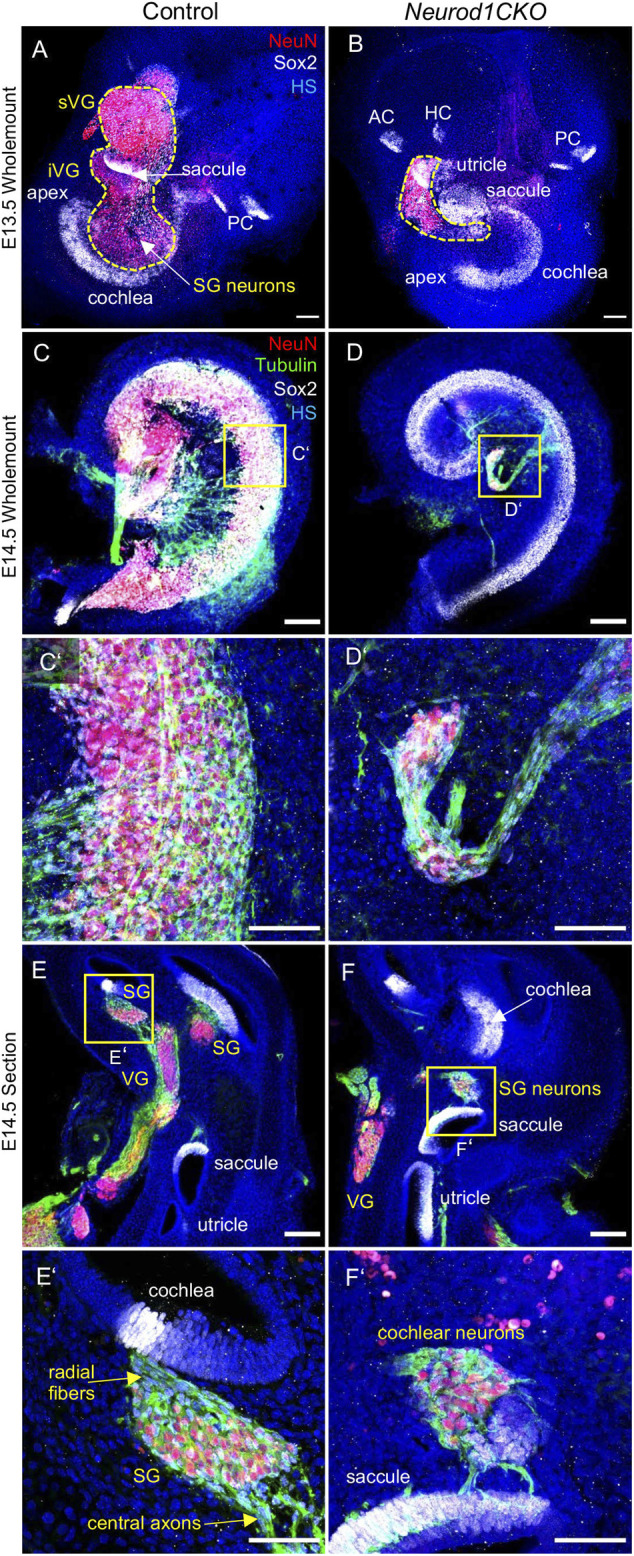
The ganglion formation in the *Neurod1CKO* cochlea is severely disrupted. **(A**,**B)** Representative images of whole-mounted inner ear labeled by anti-NeuN (a marker of differentiated neurons) and SOX2 (a marker of sensory epithelial and glial cells). The dashed line delineates the VG and SG areas containing neurons. **(C**,**D**,**C’**,**D’)** At E14.5, cochlear neurons form the SG along with the sensory cells of the organ of Corti in the control cochlea. In contrast, neurons in the *Neurod1CKO* cochlea form a rudimental ganglion located far off the organ of Corti and project unusual long fibers to the sensory epithelium. **(E**,**F)** The section of the inner ear show distribution of neurons in the VG and SG and the location of sensory epithelia. **(E’**,**F’)** Higher-magnification images show SG neurons projecting radial fibers towards the sensory epithelium and central axons in the control inner ear. In *Neurod1CKO,* cochlear neurons project aberrant fibers toward the sensory epithelium of the saccule. Scale bars: 100 μm **(A**–**F)**, 50 μm **(C**’–**F’)**. AC, anterior crista; HC, horizontal crista; HS, Hoechst nuclear staining; PC, posterior crista; SG, spiral ganglion; VG, vestibular ganglion; iVG, inferior vestibular ganglion; sVG, superior vestibular ganglion.

**FIGURE 6:**
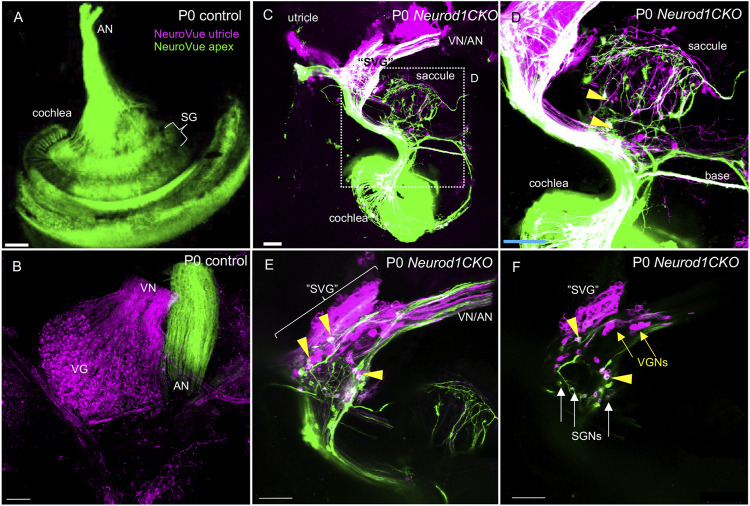
Unsegregated inner ear neurons interconnect sensory epithelia of the auditory and vestibular peripheral organs in *Neurod1CKO*. **(A**,**B)** A NeuroVue dye labeling from the utricle (magenta) and apex (green) shows restricted labeling in the cochlea, auditory nerve (AN), and vestibular ganglion neurons in the vestibular ganglion of control mice. **(C**,**D)** A similar dye application shows a unique pattern of double-labeled bilateral projections to reach saccule, base, and apex in *Neurod1CKO*. Note double-labeled neurons (arrowheads). **(E)** A larger magnification image shows the aberrant “spiro-vestibular” ganglion (“SVG”) containing a mix of neurons labeled by dyes applied to the apex and utricle in *Neurod1CKO*. Arrowheads indicate double labeled neurons with projections reaching the utricle and cochlear apex. Note unsegregated axons of vestibular and auditory neurons. **(F)** A representative one z-stack image of “SVG” shows a clutter of larger-size vestibular ganglion neurons (VGNs, arrows) with smaller-size cochlear neurons (SGNs, arrows), and double-labeled neurons (arrowheads). Scale bars: 100 µm. SG, spiral ganglion; SGNs, spiral ganglion neurons; VG, vestibular ganglion; VN, vestibular nerve.

After a period of proliferation, all inner ear neurons undergo terminal mitosis, begin to differentiate and extend their processes to the peripheral and central targets (Pavlinkova, 2020). By E13.5, sensory neurons cease to proliferate as the last spiral ganglion neurons exit the cell cycle in the apex, and thus, the final number of neurons of the mouse inner ear is established (Matei et al., 2005). The superior and inferior vestibular ganglia in the control inner ear were segregated, and the cochlear neurons formed a spiral along the sensory epithelia at E13.5 (Figure 5A). In contrast, the number of neurons in *Neurod1CKO* was severely reduced, forming a diminished vestibular ganglion and a rudiment of the cochlear ganglion near the saccule and cochlear base (Figure 5B). The abnormalities in neuronal development were even more apparent in the *Neurod1CKO* cochlea at E14.5. The spiral ganglion neurons were in the Rosenthal’s canal, extending projections, and located near the SOX2^+^ sensory epithelium in the control cochlea (Figures 5C,C’). In the *Neurod1CKO*, SOX2^+^ sensory cells formed the spiral, whereas a few residual cochlear neurons were in the modiolus away from the epithelium (Figures 5D,D’). These misplaced neurons formed projections towards the cochlea’s sensory epithelium and the saccule (Figures 5F,F’). No such projections were distinguished in the control inner ear (Figures 5E,E’). Unusual connections of these residual neurons were further explored by dye tracing analyses with dyes applied to the vestibular end-organ, the utricle, and to the cochlear apex (Figure 6). Double-labeling shows unique bundles of overlapping projections in *Neurod1CKO* (white fibers) labeled in the apex and utricle that reach the middle organ of Corti with a few branches extending to the base and the saccule (Figures 6C,D). An aberrant *Neurod1CKO* ganglion positioned next to the saccule contained double-labeled neurons projecting to the cochlear apex and the utricle. Vestibular and cochlear neurons can be identified based on their characteristic soma size. At P0, most neurons in the vestibular ganglion have an average size ∼90 μm^2^ with many of these neurons larger than 200 μm^2^, in contrast, most spiral ganglion neurons are ∼25 μm^2^ (Huang et al., 2001). A remnant of vestibular ganglion (“spiro-vestibular” ganglion) contained double-labeled neurons and a mixture of vestibular neurons (larger size soma) and cochlear neurons with noticeable smaller size soma in *Neurod1CKO* (Figures 6E,F) in contrast to the vestibular ganglion of control mice with neurons exclusively labeled by utricle-dye applications (Figure 6B).

### Elimination of *Neurod1* Affects Central Projections of Inner Ear Neurons

**FIGURE 7:**
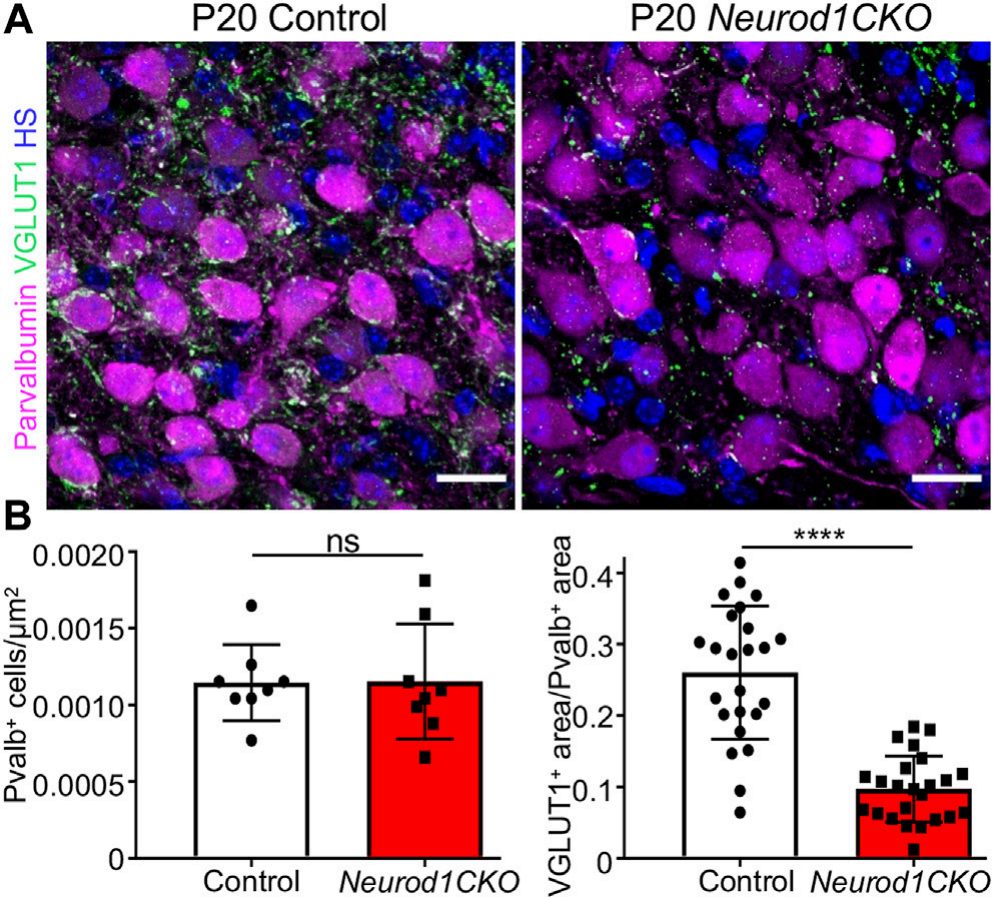
Auditory afferent synapses are reduced in the anteroventral cochlear nucleus of *Neurod1CKO* adult mice. **(A)** Representative confocal images of anti-parvalbumin labeled spherical bushy cells and anti-VGLUT1 labeled auditory-nerve endings, endbulbs of Held, show a loss of auditory afferent synapses in *Neurod1CKO* that wrap the soma of bushy cells. **(B)** Quantification of spherical bushy cells (*n* = 4 mice/two sections/genotype) and VGLUT1^+^ synapses per the parvalbumin^+^ area (*n* = 4 mice/two sections/three cells per section/genotype). The values represent means ± SD, *t-*test; ns, not significant; ****p ≤ 0.0001. Scale bars: 20 µm. HS, Hoechst nuclear staining.

**FIGURE 8:**
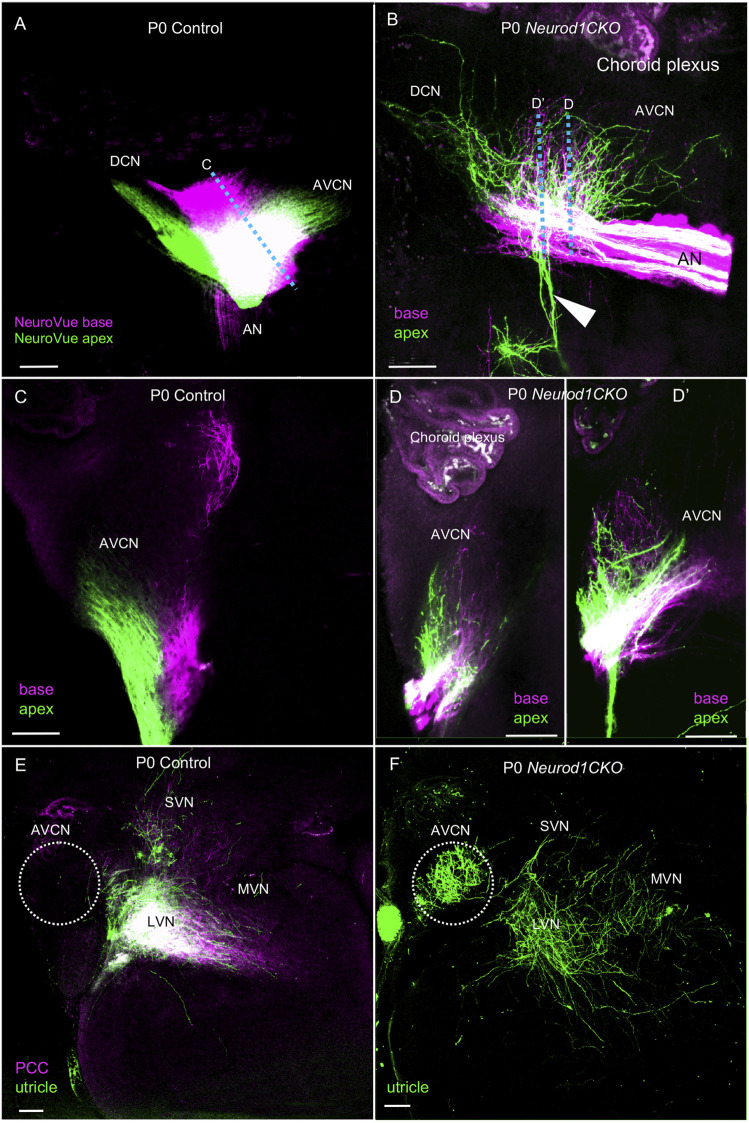
Disorganized central projections of *Neurod1CKO* inner ear neurons show aberrant interconnections between the auditory and vestibular systems. **(A**,**B)** Lipophilic differently colored NeuroVue dyes were applied to the apex and base to label cochlear afferents. In the lateral view of the cochlear nucleus of the control, dye tracing shows the normal segregated basal and apical cochlear afferents of the auditory nerve and segregated projections to the AVCN and DCN. In contrast, reduced, disorganized, and overlapping central projections are shown in the cochlear nucleus of *Neurod1CKO* mice. Note unusual fibers from the apex projecting away from the cochlear nucleus and auditory nerve (arrowhead). **(C)** Coronal sections of control show the segregation of basal and apical afferents. **(D**,**D’)** Sections of *Neurod1CKO* show an overlap of disorganized fibers from the apex and base. Dotted lines in corresponding figures indicate the section planes in **(A**,**B)**. **(E**,**F)** Dye tracing from the utricle and the posterior canal crista (PCC) shows vestibular afferents innervating the lateral, medial, and superior vestibular nuclei that are immediately adjacent to the AVCN in the control mice. A single dye tracing from the utricle shows vestibular afferents reach vestibular nuclei and form a profound projection to the AVCN in *Neurod1CKO* mice. The area of the AVCN is indicated by dotted circle. Scale bars: 100 µm. AN, auditory nerve; AVCN, anteroventral cochlear nucleus; DCN, dorsal cochlear nucleus; LVN, MVN, SVN, lateral, medial, and superior vestibular nuclei; VN, vestibular nerve.

The cochlear nucleus is the first auditory nucleus in the brain, where the auditory nerve fibers project. Although *Foxg1* ^*Cre*^ is not expressed in the cochlear nucleus (Supplementary Figure S1), a substantial size reduction of the cochlear nucleus in *Neurod1CKO* indicates a secondary effect of reduced afferent input from the cochlea (Figure 1E). In line with reduced auditory afferents, we found significantly attenuated clusters of auditory-nerve endbulbs of Held that wrap the somas of spherical bushy cells in the *Neurod1CKO* cochlear nucleus (Figure 7). A critical step in inner ear neurogenesis is the segregation of auditory and vestibular neurons into a spiral ganglion and vestibular ganglion and the segregation of the central projections (Dvorakova et al., 2020). Dye tracing analyses demonstrated disorganized and reduced central projections in the cochlear nucleus of *Neurod1CKO* compared to the segregated basal and apical projections in control mice (Figures 8A,B). Note the unusual projections from the apex near the cochlear nucleus entry area. Coronal sections of control mice showed clear segregation of the base and apex with minimal overlap (Figure 8C) in contrast to primarily overlapping and diminished basal and apical projections in the *Neurod1CKO* AVCN (Figures 8D,D’). Lipophilic NeuroVue dyes applied to the vestibular end-organs showed projections to the vestibular nuclei in the control brain (Figure 8E). Using single dye tracing, we showed that fibers labeled by dye application in the utricle reach both the cochlear nucleus (AVCN) and vestibular nuclei (LVN, SVN, MVN) in *Neurod1CKO* mice (Figure 8F), indicating an interconnection between the vestibular and auditory system.

### Ectopic Hair Cells Are Formed in the *Neurod1CKO* Inner Ear

**FIGURE 9:**
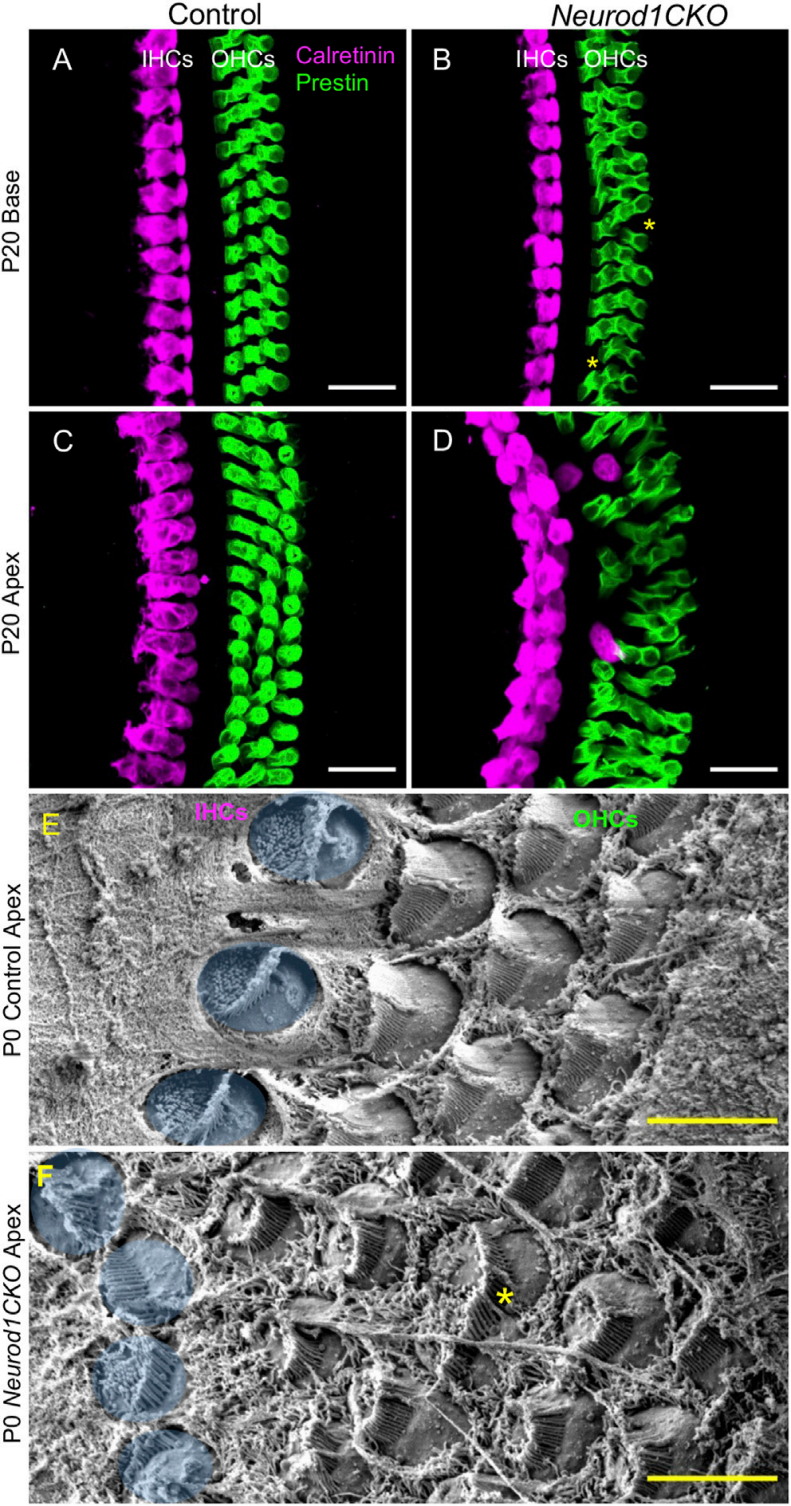
The sensory epithelium of the cochlea is disorganized in *Neurod1CKO*. **(A**–**D)** In contrast to adult controls, the sensory epithelium in the base of *Neurod1CKO* has missing cells (asterisks), as shown by confocal images of immunolabeling with inner hair cell (IHC) marker calretinin and with prestin, a marker of outer hair cells (OHCs). Ectopic (transdifferentiated) IHCs located among OHCs, two IHC rows, and multiple OHC rows are found in the *Neurod1CKO* apex compared to the single row of IHCs and three rows of OHCs in control cochlea. **(E**,**F)** Scanning electron microscopy revealed abnormalities in the cellular characteristics and hair cell stereocilia organization in *Neurod1CKO*. Compared to the control epithelium, HCs in the *Neurod1CKO* cochlea differed in their size (asterisk marks a fused cell), the orientation of cells, and stereocilia. IHCs are pseudocoloured blue. Scale bars: 20 µm **(A**–**D)**; 5 μm **(E**,**F)**.

**FIGURE 10:**
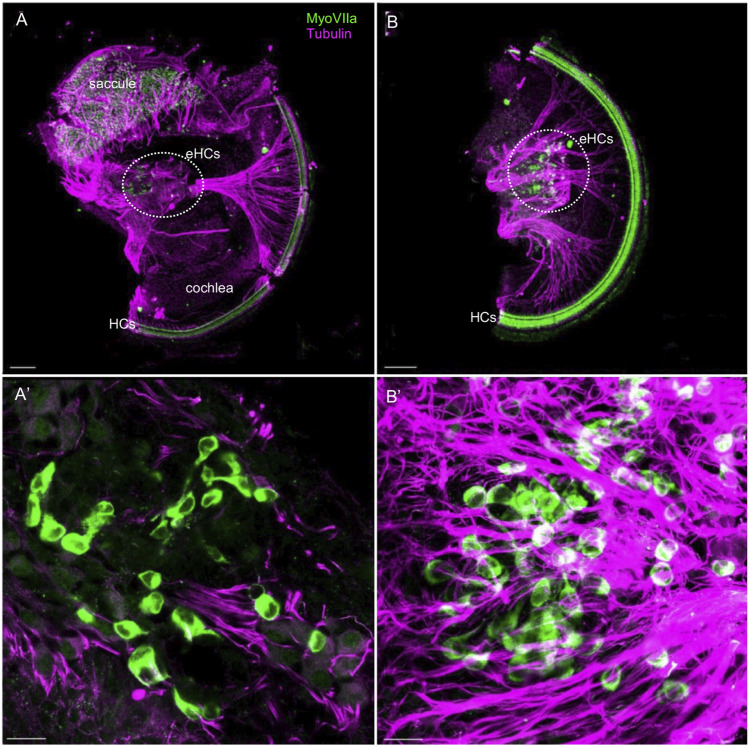
Ectopic hair cells differentiate outside the sensory epithelia in the *Neurod1CKO* inner ear. Representative images of whole mount immunolabeling with MyosinVIIa (MyoVII, a marker of hair cells) and tubulin (neuronal fibers) show hair cells in the saccule and rows of hair cells forming the organ of Corti in the *Neurod1CKO* cochlea. Ectopic MyoVIIa positive cells (eHCs) are found near the saccule and the aberrant cochlear ganglion (a dotted line delineates the area). Larger magnification images show eHCs entangled with neuronal fibers, and some cells are double-labeled for tubulin and MyoVIIa. Scale bars: 100 µm **(A**,**B)**; 20 μm **(A’**,**B’)**.

The development of sensory cells was disrupted by the elimination of *Neurod1,* indicated by significantly smaller vestibular end-organ sensory epithelia and a shorter organ of Corti in the cochlea (Figure 2 and Supplementary Figure S5). Additionally, the cochlear sensory epithelium was disorganized. Immunolabeling with the inner hair cell (IHC) marker calretinin and prestin, a marker of outer hair cells (OHC), showed multiple OHC rows, transdifferentiated (ectopic) IHCs among OHCs, and two rows of IHCs in the adult apex of *Neurod1CKO* compared to three rows of OHCs and a single row of IHCs in control (Figures 9C,D). Missing OHCs were found in the *Neurod1CKO* base (Figures 9A,B). Scanning electron microscopy images demonstrated cellular abnormalities in the sensory epithelium of *Neurod1CKO*, indicating disrupted planar cell polarity (Figures 9E,F). An additional effect associated with *Neurod1* deficiency was a noticeable presence of ectopic hair cells located outside of sensory epithelia. Besides regular sensory epithelia containing MyosinVIIa expressing hair cells in the saccule, and IHCs and OHCs in the organ of Corti *Neurod1CKO* cochlea, extra ectopic MyosinVIIa positive cells were found near the saccule (Figure 10A and Supplementary Figure S5B) and in the cochlea, near the rudimental spiral ganglion (Figures 10A,B). Detailed images showed the distribution of ectopic hair cells intermingled with neuronal fibers, and some cells were double labeled by tubulin and MyosinVIIa (Figures 10A’,B’). These topologically inappropriate “trans-fated” ectopic hair cells form a unique organization in the area of the two neuronal aggregations of vestibular and cochlear neurons, independent of known inner ear structures.

## Discussion

**FIGURE 11:**
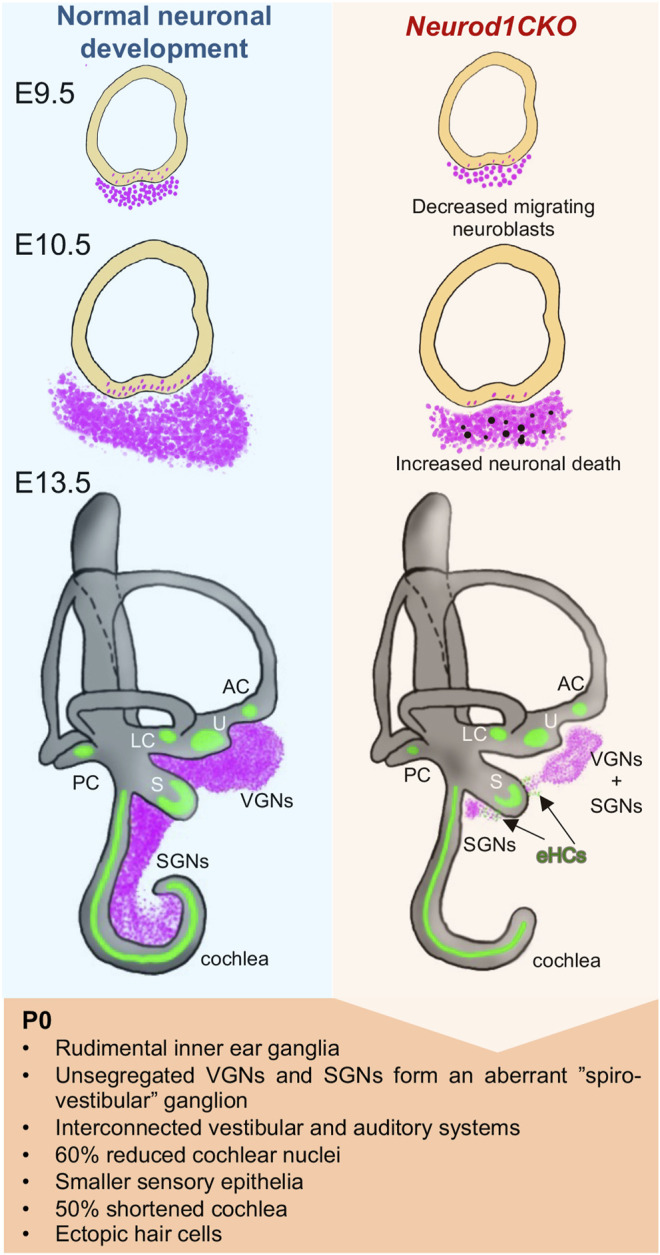
Schematics of changes induced by the elimination of *Neurod1* in the inner ear. Absence of *Neurod1* results in reduced delamination and increased apoptosis of neuroblasts at E10.5. Remaining post-mitotic neurons form a misplaced rudimental spiral ganglion and diminished vestibular ganglion (“spiro-vestibular” ganglion), containing unsegregated vestibular and cochlear neurons that extend unsegregated peripheral and central projections, resulting in circuits interconnecting peripheral sensory organs and brain nuclei of both the auditory and vestibular systems. The reduced afferent input from the cochlea affects the size of the cochlear nucleus of the brain stem in *Neurod1CKO* mice. Additionally, *Neurod1* elimination results in smaller sensory epithelia, a loss of more than the half size of the cochlea, disorganization of sensory cells, multiple rows of inner and outer hair cells, trans-differentiation of outer hair cells in the apex, and generation of ectopic hair cells (eHCs) in the areas of inner ear ganglia.

The focus of this study was to determine the requirements for NEUROD1 in the early neuronal development of the inner ear and processes that are affected by *Neurod1* elimination (Figure 11). NEUROD1, a bHLH transcription factor, is an essential factor for the lineage commitment and differentiation in various developmental systems, including gastrointestinal cells (Cherry et al., 2011), pancreas (Romer et al., 2019; Bohuslavova et al., 2021), brain (Miyata et al., 1999; Liu et al., 2000b; Hevner et al., 2006), and neurosensory organs (Liu et al., 2000a; Cherry et al., 2011). The main function of NEUROD1 in neurogenesis and the promotion of neuronal fate is supported by its ability to reprogram other somatic cells into neurons. For example, forced expression of *Neurod1* enhances neuronal conversion from human fibroblasts (Pang et al., 2011), and exogenous *Neurod1* expression converts mouse astrocytes to neurons (Guo et al., 2014). In the inner ear, neuronal development is initiated by the upregulation of *Neurog1* in the anteroventral quadrant of the otocyst. *Neurog1* is followed by the upregulation of *Neurod1* in the proneural domain of the otocyst, resulting in the delamination and migration of primary neuroblasts to the forming otic ganglion. Delaminating neuronal progenitors proliferate and differentiate to produce vestibular and auditory neurons between E9.5 to E13.5 (Matei et al., 2005).

In contrast to the *Neurog1* deletion mutant with no neurons found in the E10.5 otic ganglion (Ma et al., 1998) or any later stages of inner ear development (Liberman, 1991), some inner ear neurons differentiate and form peripheral and central projections in *Neurod1* null mice (Liu et al., 2000a; Kim et al., 2001). Previously, we generated a delayed *Neurod1* conditional deletion using *Isl1* ^*Cre*^ that spares many cochlear and vestibular neurons, resulting in the near-normal vestibular behavior and partially preserved auditory system function (Macova et al., 2019). Similarly, *Pax2* ^*Cre*^ is delayed and leaves many more neurons in conditional *Neurod1* deletion mice (Jahan et al., 2010a) relative to *Foxg1* ^*Cre*^, used in this study. Consequently, these phenotypes indicate a temporal effect of *Neurod1* elimination for the neuronal loss in the developing inner ear. However, mechanisms of neuronal loss due to the *Neurod1* elimination are not fully understood. Different mechanisms have been implicated in determining neuron quantities, including neuronal delamination, migration, proliferation, and survival. Our study revisited the major events of early neuronal development of the inner ear to address different regulatory functions and temporal requirements for NEUROD1.

Foxg1^Cre^ recombination occurs efficiently and invariably in the ear placode, forebrain, olfactory and pre-lens placodes, anterior optic vesicle, pharyngeal endoderm, and mid-hindbrain junction, matching the normal pattern of *Foxg1* expression (Dvorakova et al., 2020; Hébert and McConnell, 2000; Dastidar et al., 2011; Duggan et al., 2008; Shen et al., 2018; Panaliappan et al., 2018). A caveat must be considered in using *Foxg1* ^*Cre*^ line. First, haploinsufficiency of the *Foxg1* gene due to a partial replacement of *Foxg1* coding sequences with the cre gene (Hébert and McConnell, 2000) is associated with impaired development of the telencephalon and microcephaly in heterozygous *Foxg1* ^*Cre/+*^ transgenic mice similar to the phenotype of heterozygous *Foxg1* ^*−/+*^ null mice (Shen et al., 2018; Frullanti et al., 2016; Eagleson et al., 2007; Kawaguchi et al., 2016). Correspondingly, a similar neurodevelopmental phenotype of a reduced size of the forebrain was found in our heterozygous *Foxg1* ^*Cre/+*^ *, Neurod1* ^*loxP/+*^ mutants (Figure 1). Second, ectopic low levels of Cre activity have been reported due to genetic background effects (Hébert and McConnell, 2000; Ma et al., 2002). To confirm the scope of Cre activity in our model, we crossed *Foxg1* ^*Cre*^ mice to a *tdTomatoAi14* reporter mouse strain. We verified Foxg1^Cre^-mediated loxP recombination in the organ of Corti, spiral ganglion neurons, and the cerebellum of adult progeny recapitulating endogenous *Foxg1* expression (Supplementary Figure S1). We did not detect any Foxg1^Cre^ activity in the cochlear nucleus, showing no tdTomato expression in neurons of the cochlear nucleus. Because *Foxg1* ^*Cre*^ is not expressed in the cochlear nucleus, the reduced size of the *Neurod1CKO* cochlear nucleus is exclusively an effect of diminished afferent input from spiral ganglion type I neurons, similar to cochlear ablation studies (Mostafapour et al., 2000).

We showed that early elimination of *Neurod1* affected delaminating and migrating ISL1^+^ neurons in the E9.5 and E10.5, resulting in a significantly smaller inner ear ganglion at E10.5 (Figure 4). One mechanism contributing to the diminished number of inner ear neurons in *Neurod1CKO* was massive apoptosis at E10.5, confirming that *Neurod1* is required for early neuronal survival (Liu et al., 2000a). In contrast, neuroblasts lacking *Neurod1* proliferate at a similar rate as neuronal precursors in control embryos, suggesting that NEUROD1 is not needed for the proliferation of neurons, at least in the E10.5 inner ear. Intriguingly, our data confirmed the initial finding in the global *Neurod1* deletion mutant (Liu et al., 2000a) that some inner ear neurons survive without *Neurod1*. One could speculate that the neurogenic fate commitment is predefined early in some Neurog1^+^ specified neuronal precursors ensuring terminal differentiation of these *Neurod1* null neurons. Alternatively, residual neurons might survive by compensatory activation of different transcription factor(s), such as GATA3 (Duncan and Fritzsch, 2013) or NHLH1/NHLH2 bHLH factors (Krüger et al., 2006).

Despite diminished neurogenesis, these *Neurod1* lacking neurons form inner ear ganglia, grow neuritic processes, establish bipolar connections to their targets, and persist up to the adulthood of *Neurod1CKO* mice. The formation of inner ear ganglia was considerably disrupted when the last sensory neurons undergo terminal mitosis, at E13.5 (Matei et al., 2005). The vestibular ganglion was diminished, and the spiral ganglion was represented by a small clump of neurons near the saccule in the *Neurod1CKO* inner ear (Figure 5). In addition to a significant loss of neurons, the rudiment of the spiral ganglion was misplaced away from the sensory epithelium in the *Neurod1CKO* cochlea, indicating migration defects. Cochlear neurons found in the aberrant vestibular ganglion (“spiro-vestibular” ganlion) of *Neurod1CKO* further corroborate abnormalities in inner ear neurons’ migration (Figure 6). The remaining neurons projected disorganized peripheral fibers, with some neurons forming unusual peripheral projections connecting the cochlea and the vestibular end-organs. The central projections of our *Neurod1CKO* mutant are primarily identical to central projection abnormalities in various *Neurod1* deletion mutants (Kim et al., 2001; Jahan et al., 2010a; Macova et al., 2019; Filova et al., 2020). Mainly, central projections of cochlear and vestibular neurons are reduced, unsegregated, and disorganized. Using our *Neurod1* mutant, we showed, for the first time, central projections from vestibular neurons reached the cochlear nucleus, the first processing station in the central auditory system (Figure 8). Consistently with the aberrant migration and disorganized innervation phenotypes of *Neurod1* mutants, NEUROD1 orchestrates transcriptional networks regulating axon guidance, neurite growth, cell migration, and adhesion in post-mitotic inner ear neurons (Filova et al., 2020).

In addition to neuronal phenotypes, *Neurod1* deletion affects the development of inner ear sensory organs (Kim et al., 2001; Macova et al., 2019; Filova et al., 2020; Jahan et al., 2010b). Like other *Neurod1* deletion mutants, due to *Foxg1* ^*Cre*^ activity in the sensory precursors, we were unable to uncouple secondary effects of *Neurod1* deletion in inner ear neurons on sensory cell development. The elimination of *Neurod1* affects the size of inner ear sensory epithelia and the differentiation and organization of apical epithelium in the cochlea. Nevertheless, some effects are more pronounced in our *Neurod1CKO* than in delayed *Neurod1* mutants suggesting a temporal dependency of *Neurod1* elimination. Particularly, the truncated cochlea of *Neurod1CKO* reached 47% length of the control cochlea compared to 60% of the cochlea of mice with delayed *Neurod1* mutation (Macova et al., 2019). We also found a more profound formation of ectopic myosin VIIa positive hair cells near the two remaining neuronal aggregations of vestibular and cochlear sensory neurons (Figure 10) compared to the *Neurod1* conditional mutation generated by *Pax2* ^*Cre*^ (Jahan et al., 2010b). Previous work identified a set of upregulated genes in the absence of *Neurod1*, particularly *Atoh1*, *Fgf8,* and *Nhlh2* (Jahan et al., 2010b), suggesting that some neuronal precursors adopt a hair cell fate and differentiate as hair cells (Fritzsch and Elliott, 2017; Elliott et al., 2021). Taken together, NEUROD1 mediates a neuronal program and promotes neuronal fate by the upregulation of downstream targets and through suppression of other bHLH genes such as *Atoh1*. Another possible role of NEUROD1 in neurogenesis and neurogenic competence may be reprograming of the epigenome in the developing inner ear (Matsuda et al., 2019). Further experiments will be necessary to investigate how NEUROD1 mediates transcriptional and possibly epigenetic networks during neuronal development in the inner ear. By understanding the interplay of NEUROD1 regulatory networks capable of initiating cell-fate changes, we hope to develop efficient therapeutic strategies to restore and regenerate neurons for clinical applications.

A crucial role of NEUROD1 in neurogenesis and neuronal differentiation in the inner ear is conserved across vertebrates, as shown by studies in chicken (Evsen et al., 2013), *Xenopus laevis* (Schlosser and Northcutt, 2000), and zebrafish (Schwarzer et al., 2020). These studies also provide significant insights into the role of NEUROD1 as a neurogenic programing factor. For example, ectopic expression of *Neurod1* is sufficient to mediate neurogenesis in the chicken developing inner ear (Evsen et al., 2013). In zebrafish, the expression of *Neurod1* is maintained in progenitor cells in a neurogenic niche that are expected to contribute to neuronal regeneration in the adult zebrafish statoacoustic ganglion (Schwarzer et al., 2020). Understanding a role of NEUROD1 in neurogenesis of species that can produced neurons throughout life and regenerate damaged neurons can provide significant insights into the rekindling of regeneration in the mammalian inner ear. An importance of NEUROD1 as a neurogenic reprograming factor is supported by *in vivo* studies utilizing *Neurod1* to produce functional neurons by reprograming astrocytes (Guo et al., 2014; Brulet et al., 2017). A reprogramming cells within the auditory system is considered a promising way to cure hearing loss (Meas et al., 2018).

## Concluding Remarks

Our findings provide insights into the temporal requirements for NEUROD1 during inner ear development. In early neuronal development, we demonstrated that NEUROD1 is necessary for the generation and survival of neuronal precursors, as the absence of *Neurod1* results in massive apoptosis of E10.5 neuroblasts. In later neuronal development of post-mitotic neurons, *Neurod1* is critical for segregating neurons of the vestibular and auditory systems, migration, and formation of peripheral and central projections of these neurons. Understanding how neuronal fate is promoted within the nervous system and how neural circuit assemblies are organized has essential implications for cell-based therapy.

## Data Availability

The original contributions presented in the study are included in the article/Supplementary Material, further inquiries can be directed to the corresponding author.

## References

[B1] Abrams S. R. Reiter J. F. (2021). Ciliary Hedgehog Signaling Regulates Cell Survival to Build the Facial Midline. Elife 10, e68558. 10.7554/elife.68558 34672258 PMC8592574

[B2] Bermingham N. A. Hassan B. A. Price S. D. Vollrath M. A. Ben-Arie N. Eatock R. A. (1999). Math1 : An Essential Gene for the Generation of Inner Ear Hair Cells. Science 284, 1837–1841. 10.1126/science.284.5421.1837 10364557

[B3] Bérubé N. G. Mangelsdorf M. Jagla M. Vanderluit J. Garrick D. Gibbons R. J. (2005). The Chromatin-Remodeling Protein ATRX Is Critical for Neuronal Survival During Corticogenesis. J. Clin. Invest. 115, 258–267. 10.1172/jci200522329 15668733 PMC544602

[B4] Bohuslavova R. Smolik O. Malfatti J. Berkova Z. Novakova Z. Saudek F. (2021). NEUROD1 Is Required for the Early Alpha and Beta Endocrine Differentiation in the Pancreas. Int. J. Mol. Sci. 22, 6713. 10.3390/ijms22136713 34201511 PMC8268837

[B5] Brulet R. Matsuda T. Zhang L. Miranda C. Giacca M. Kaspar B. K. (2017). NEUROD1 Instructs Neuronal Conversion in Non-reactive Astrocytes. Stem Cel Rep. 8, 1506–1515. 10.1016/j.stemcr.2017.04.013 PMC5470076 28506534

[B6] Cherry T. J. Wang S. Bormuth I. Schwab M. Olson J. Cepko C. L. (2011). NeuroD Factors Regulate Cell Fate and Neurite Stratification in the Developing Retina. J. Neurosci. 31, 7365–7379. 10.1523/jneurosci.2555-10.2011 21593321 PMC3135085

[B7] Dastidar S. G. Landrieu P. M. Z. D'Mello S. R. (2011). FoxG1 Promotes the Survival of Postmitotic Neurons. J. Neurosci. 31, 402–413. 10.1523/jneurosci.2897-10.2011 21228151 PMC4640444

[B8] Duggan C. D. DeMaria S. Baudhuin A. Stafford D. Ngai J. (2008). Foxg1 Is Required for Development of the Vertebrate Olfactory System. J. Neurosci. 28, 5229–5239. 10.1523/jneurosci.1134-08.2008 18480279 PMC2706027

[B9] Duncan J. S. Fritzsch B. (2013). Continued Expression of GATA3 Is Necessary for Cochlear Neurosensory Development. PLoS One 8, e62046. 10.1371/journal.pone.0062046 23614009 PMC3628701

[B10] Dvorakova M. Macova I. Bohuslavova R. Anderova M. Fritzsch B. Pavlinkova G. (2020). Early Ear Neuronal Development, but Not Olfactory or Lens Development, Can Proceed Without SOX2. Dev. Biol. 457, 43–56. 10.1016/j.ydbio.2019.09.003 31526806 PMC6938654

[B11] Eagleson K. L. Schlueter McFadyen-Ketchum L. J. Ahrens E. T. Mills P. H. Does M. D. Nickols J. (2007). Disruption of Foxg1 Expression by Knock-In of Cre Recombinase: Effects on the Development of the Mouse Telencephalon. Neuroscience 148, 385–399. 10.1016/j.neuroscience.2007.06.012 17640820 PMC2194757

[B12] Elliott K. L. Fritzsch B. Duncan J. S. (2018). Evolutionary and Developmental Biology Provide Insights into the Regeneration of Organ of Corti Hair Cells. Front Cel Neurosci 12, 252. 10.3389/fncel.2018.00252 PMC6092489 30135646

[B13] Elliott K. L. Pavlínková G. Chizhikov V. V. Yamoah E. N. Fritzsch B. (2021). Development in the Mammalian Auditory System Depends on Transcription Factors. Ijms 22, 4189. 10.3390/ijms22084189 33919542 PMC8074135

[B14] Evsen L. Sugahara S. Uchikawa M. Kondoh H. Wu D. K. (2013). Progression of Neurogenesis in the Inner Ear Requires Inhibition of Sox2 Transcription by Neurogenin1 and Neurod1. J. Neurosci. 33, 3879–3890. 10.1523/jneurosci.4030-12.2013 23447599 PMC3865497

[B15] Filova I. Dvorakova M. Bohuslavova R. Pavlinek A. Elliott K. L. Vochyanova S. (2020). Combined Atoh1 and Neurod1 Deletion Reveals Autonomous Growth of Auditory Nerve Fibers. Mol. Neurobiol. 57, 5307–5323. 10.1007/s12035-020-02092-0 32880858 PMC7547283

[B16] Fritzsch B. Duncan J. S. Kersigo J. Gray B. Elliott K. L. (2016). Neuroanatomical Tracing Techniques in the Ear: History, State of the Art, and Future Developments. Springer, 243–262. 10.1007/978-1-4939-3615-1_14 PMC4993453 27259931

[B17] Fritzsch B. Elliott K. L. (2017). Gene, Cell, and Organ Multiplication Drives Inner Ear Evolution. Dev. Biol. 431, 3–15. 10.1016/j.ydbio.2017.08.034 28866362 PMC5643246

[B18] Fritzsch B. Pan N. Jahan I. Duncan J. S. Kopecky B. J. Elliott K. L. (2013). Evolution and Development of the Tetrapod Auditory System: An Organ of Corti-Centric Perspective. Evol. Dev. 15, 63–79. 10.1111/ede.12015 23331918 PMC3918746

[B19] Fritzsch B. Straka H. (2014). Evolution of Vertebrate Mechanosensory Hair Cells and Inner Ears: Toward Identifying Stimuli that Select Mutation Driven Altered Morphologies. J. Comp. Physiol. A. 200, 5–18. 10.1007/s00359-013-0865-z PMC3918741 24281353

[B20] Frullanti E. Amabile S. Lolli M. G. Bartolini A. Livide G. Landucci E. (2016). Altered Expression of Neuropeptides in FoxG1-Null Heterozygous Mutant Mice. Eur. J. Hum. Genet. 24, 252–257. 10.1038/ejhg.2015.79 25966633 PMC4717204

[B21] Goebbels S. Bode U. Pieper A. Funfschilling U. Schwab M. H. Nave K.-A. (2005). Cre/loxP-mediated Inactivation of the bHLH Transcription Factor Gene NeuroD/BETA2. Genesis 42, 247–252. 10.1002/gene.20138 16028233

[B22] Goodrich L. V. (2016). Early Development of the Spiral Ganglion, the Primary Auditory Neurons of the Mammalian Cochlea. Springer 1, 11–48. 10.1007/978-1-4939-3031-9_2

[B23] Guo Z. Zhang L. Wu Z. Chen Y. Wang F. Chen G. (2014). In Vivo Direct Reprogramming of Reactive Glial Cells into Functional Neurons After Brain Injury and in an Alzheimer's Disease Model. Cell Stem Cell 14, 188–202. 10.1016/j.stem.2013.12.001 24360883 PMC3967760

[B24] Hébert J. M. McConnell S. K. (2000). Targeting of Cre to the Foxg1 (BF-1) Locus Mediates loxP Recombination in the Telencephalon and Other Developing Head Structures. Dev. Biol. 222, 296–306. 10.1006/dbio.2000.9732 10837119

[B25] Hevner R. F. Hodge R. D. Daza R. A. M. Englund C. (2006). Transcription Factors in Glutamatergic Neurogenesis: Conserved Programs in Neocortex, Cerebellum, and Adult hippocampus. Neurosci. Res. 55, 223–233. 10.1016/j.neures.2006.03.004 16621079

[B26] Huang E. J. Liu W. Fritzsch B. Bianchi L. M. Reichardt L. F. Xiang M. (2001). Brn3a Is a Transcriptional Regulator of Soma Size, Target Field Innervation and Axon Pathfinding of Inner Ear Sensory Neurons. Development 128, 2421–2432. 10.1242/dev.128.13.2421 11493560 PMC2710107

[B27] Jahan I. Kersigo J. Pan N. Fritzsch B. (2010). Neurod1 Regulates Survival and Formation of Connections in Mouse Ear and Brain. Cell Tissue Res 341, 95–110. 10.1007/s00441-010-0984-6 20512592 PMC3657738

[B28] Jahan I. Pan N. Kersigo J. Fritzsch B. (2010). Neurod1 Suppresses Hair Cell Differentiation in Ear Ganglia and Regulates Hair Cell Subtype Development in the Cochlea. PLoS One 5, e11661. 10.1371/journal.pone.0011661 20661473 PMC2908541

[B29] Kasberg A. D. Brunskill E. W. Steven Potter S. (2013). SP8 Regulates Signaling Centers During Craniofacial Development. Dev. Biol. 381, 312–323. 10.1016/j.ydbio.2013.07.007 23872235 PMC4078980

[B30] Kawaguchi D. Sahara S. Zembrzycki A. O’Leary D. D. M. (2016). Generation and Analysis of an Improved Foxg1-IRES-Cre Driver Mouse Line. Dev. Biol. 412, 139–147. 10.1016/j.ydbio.2016.02.011 26896590 PMC5895454

[B31] Khan S. Chang R. (2013). Anatomy of the Vestibular System: A Review. Nre 32, 437–443. 10.3233/nre-130866 23648598

[B32] Kim W. Y. Fritzsch B. Serls A. Bakel L. A. Huang E. J. Reichardt L. F. (2001). NeuroD-null Mice Are Deaf Due to a Severe Loss of the Inner Ear Sensory Neurons During Development. Development 128, 417–426. 10.1242/dev.128.3.417 11152640 PMC2710102

[B33] Krüger M. Schmid T. Krüger S. Bober E. Braun T. (2006). Functional Redundancy of NSCL-1 and NeuroD during Development of the Petrosal and Vestibulocochlear Ganglia. Eur. J. Neurosci. 24, 1581–1590. 10.1111/j.1460-9568.2006.05051.x 17004922

[B34] Liberman M. C. (1991). The Olivocochlear Efferent Bundle and Susceptibility of the Inner Ear to Acoustic Injury. J. Neurophysiol. 65, 123–132. 10.1152/jn.1991.65.1.123 1999726

[B35] Liu M. Pereira F. A. Price S. D. Chu M.-j. Shope C. Himes D. (2000). Essential Role of BETA2/NeuroD1 in Development of the Vestibular and Auditory Systems. Genes Dev. 14, 2839–2854. 10.1101/gad.840500 11090132 PMC317056

[B36] Liu M. Pleasure S. J. Collins A. E. Noebels J. L. Naya F. J. Tsai M.-J. (2000). Loss of BETA2/NeuroD Leads to Malformation of the Dentate Gyrus and Epilepsy. Proc. Natl. Acad. Sci. 97, 865–870. 10.1073/pnas.97.2.865 10639171 PMC15422

[B37] Ma L. Harada T. Harada C. Romero M. Hebert J. M. McConnell S. K. (2002). Neurotrophin-3 Is Required for Appropriate Establishment of Thalamocortical Connections. Neuron 36, 623–634. 10.1016/s0896-6273(02)01021-8 12441052

[B38] Ma Q. Anderson D. J. Fritzsch B. (2000). Neurogenin 1 Null Mutant Ears Develop Fewer, Morphologically Normal Hair Cells in Smaller Sensory Epithelia Devoid of Innervation. Jaro 1, 129–143. 10.1007/s101620010017 11545141 PMC2504536

[B39] Ma Q. Chen Z. Barrantes I. d. B. Luis de la Pompa J. Anderson D. J. (1998). neurogenin1 Is Essential for the Determination of Neuronal Precursors for Proximal Cranial Sensory Ganglia. Neuron 20, 469–482. 10.1016/s0896-6273(00)80988-5 9539122

[B40] Macova I. Pysanenko K. Chumak T. Dvorakova M. Bohuslavova R. Syka J. (2019). Neurod1 Is Essential for the Primary Tonotopic Organization and Related Auditory Information Processing in the Midbrain. J. Neurosci. 39, 984–1004. 10.1523/jneurosci.2557-18.2018 30541910 PMC6363931

[B41] Matei V. Pauley S. Kaing S. Rowitch D. Beisel K. W. Morris K. (2005). Smaller Inner Ear Sensory Epithelia in Neurog1 Null Mice Are Related to Earlier Hair Cell Cycle Exit. Dev. Dyn. 234, 633–650. 10.1002/dvdy.20551 16145671 PMC1343505

[B42] Matsuda T. Irie T. Katsurabayashi S. Hayashi Y. Nagai T. Hamazaki N. (2019). Pioneer Factor NeuroD1 Rearranges Transcriptional and Epigenetic Profiles to Execute Microglia-Neuron Conversion. Neuron 101, 472–485. 10.1016/j.neuron.2018.12.010 30638745

[B43] Meas S. J. Zhang C.-L. Dabdoub A. (2018). Reprogramming Glia into Neurons in the Peripheral Auditory System as a Solution for Sensorineural Hearing Loss: Lessons from the Central Nervous System. Front. Mol. Neurosci. 11, 77. 10.3389/fnmol.2018.00077 29593497 PMC5861218

[B44] Miyata T. Maeda T. Lee J. E. (1999). NeuroD Is Required for Differentiation of the Granule Cells in the Cerebellum and hippocampus. Genes Dev. 13, 1647–1652. 10.1101/gad.13.13.1647 10398678 PMC316850

[B45] Mostafapour S. P. Cochran S. L. Del Puerto N. M. Rubel E. W. (2000). Patterns of Cell Death in Mouse Anteroventral Cochlear Nucleus Neurons after Unilateral Cochlea Removal. J. Comp. Neurol. 426, 561–571. 10.1002/1096-9861(20001030)426::4<561aid-cne5>3.0.co;2-g 11027399

[B46] Packard A. Giel-Moloney M. Leiter A. Schwob J. E. (2011). Progenitor Cell Capacity of NeuroD1-Expressing Globose Basal Cells in the Mouse Olfactory Epithelium. J. Comp. Neurol. 519, 3580–3596. 10.1002/cne.22726 21800309 PMC4005605

[B47] Panaliappan T. K. Wittmann W. Jidigam V. K. Mercurio S. Bertolini J. A. Sghari S. (2018). Sox2 Is Required for Olfactory Pit Formation and Olfactory Neurogenesis through BMP Restriction and Hes5 Upregulation. Development 145, dev153791. 10.1242/dev.153791 29352015 PMC5825848

[B48] Pang Z. P. Yang N. Vierbuchen T. Ostermeier A. Fuentes D. R. Yang T. Q. (2011). Induction of Human Neuronal Cells by Defined Transcription Factors. Nature 476, 220–223. 10.1038/nature10202 21617644 PMC3159048

[B49] Pavlinkova G. (2020). Molecular Aspects of the Development and Function of Auditory Neurons. Int. J. Mol. Sci. 22, 131. 10.3390/ijms22010131 PMC7796308 33374462

[B50] Pennesi M. E. Cho J.-H. Yang Z. Wu S. H. Zhang J. Wu S. M. (2003). BETA2/NeuroD1 Null Mice: A New Model for Transcription Factor-dependent Photoreceptor Degeneration. J. Neurosci. 23, 453–461. 10.1523/jneurosci.23-02-00453.2003 12533605 PMC6741880

[B51] Romer A. I. Singer R. A. Sui L. Egli D. Sussel L. (2019). Murine Perinatal β-Cell Proliferation and the Differentiation of Human Stem Cell-Derived Insulin-Expressing Cells Require NEUROD1. Diabetes 68, 2259–2271. 10.2337/db19-0117 31519700 PMC6868472

[B52] Rubel E. W. Fritzsch B. (2002). Auditory System Development: Primary Auditory Neurons and Their Targets. Annu. Rev. Neurosci. 25, 51–101. 10.1146/annurev.neuro.25.112701.142849 12052904

[B53] Schlosser G. Northcutt R. G. (2000). Development of Neurogenic Placodes inXenopus Laevis. J. Comp. Neurol. 418, 121–146. 10.1002/(sici)1096-9861(20000306)418::2<121aid-cne1>3.0.co;2-m 10701439

[B54] Schmidt H. Fritzsch B. (2019). Npr2 Null Mutants Show Initial Overshooting Followed by Reduction of Spiral Ganglion Axon Projections Combined with Near-normal Cochleotopic Projection. Cel Tissue Res 378, 15–32. 10.1007/s00441-019-03050-6 PMC7243364 31201541

[B55] Schwarzer S. Asokan N. Bludau O. Chae J. Kuscha V. Kaslin J. (2020). Correction: Neurogenesis in the Inner Ear: the Zebrafish Statoacoustic Ganglion Provides New Neurons from a Neurod/Nestin-Positive Progenitor Pool Well into Adulthood. Development 147, 7. 10.1242/dev.191775 32165493

[B56] Shen W. Ba R. Su Y. Ni Y. Chen D. Xie W. (2018). Foxg1 Regulates the Postnatal Development of Cortical Interneurons. Cereb. Cortex 29(4):1547. 10.1093/cercor/bhy051 PMC6676970 29912324

[B57] Wu D. K. Kelley M. W. (2012). Molecular Mechanisms of Inner Ear Development. Cold Spring Harbor Perspect. Biol. 4, a008409. 10.1101/cshperspect.a008409 PMC3405860 22855724

