# Supplementary material for: Early Deletion of Neurod1 Alters Neuronal Lineage Potential and Diminishes Neurogenesis in the Inner Ear
Source: Front Cell Dev Biol. 2022 Feb 17;10:845461. doi: 10.3389/fcell.2022.845461 (PMC8894106; doi:10.3389/fcell.2022.845461)
Supplement: Supplementary file 1 [file Presentation1.PPTX]

## Slide 1
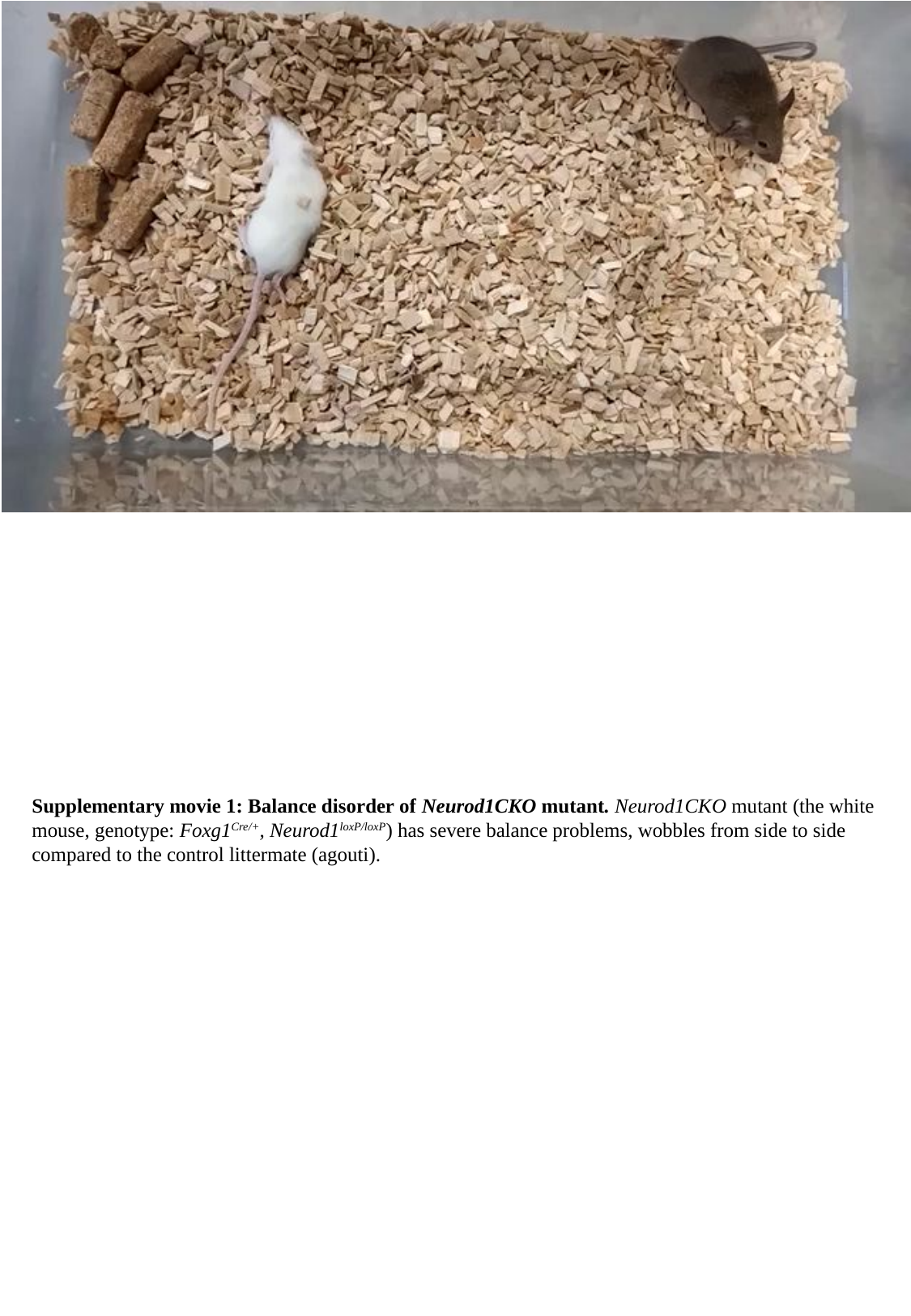

Supplementary movie 1: Balance disorder of Neurod1CKO mutant. Neurod1CKO mutant (the white mouse, genotype: Foxg1Cre/+, Neurod1loxP/loxP) has severe balance problems, wobbles from side to side compared to the control littermate (agouti).
