# Supplementary material for: Early Deletion of Neurod1 Alters Neuronal Lineage Potential and Diminishes Neurogenesis in the Inner Ear
Source: Front Cell Dev Biol. 2022 Feb 17;10:845461. doi: 10.3389/fcell.2022.845461 (PMC8894106; doi:10.3389/fcell.2022.845461)
Supplement: Supplementary file 2 [file DataSheet1.PDF]

Supplementary Material

Early Deletion of *Neurod1* Alters Neuronal Lineage Potential and Diminishes Neurogenesis in the Inner Ear

Iva Filova[1], Romana Bohuslavova[1], Mitra Tavakoli[1], Ebenezer N. Yamoah[2], Bernd Fritzsch[3], Gabriela Pavlinkova[1*]

[1]Laboratory of Molecular Pathogenesis, Institute of Biotechnology CAS, Vestec, Czechia,

[2]Department of Physiology and Cell Biology, Institute for Neuroscience, University of Nevada, Reno, NV, USA

[3]Department of Biology, University of Iowa, Iowa City, IA, USA

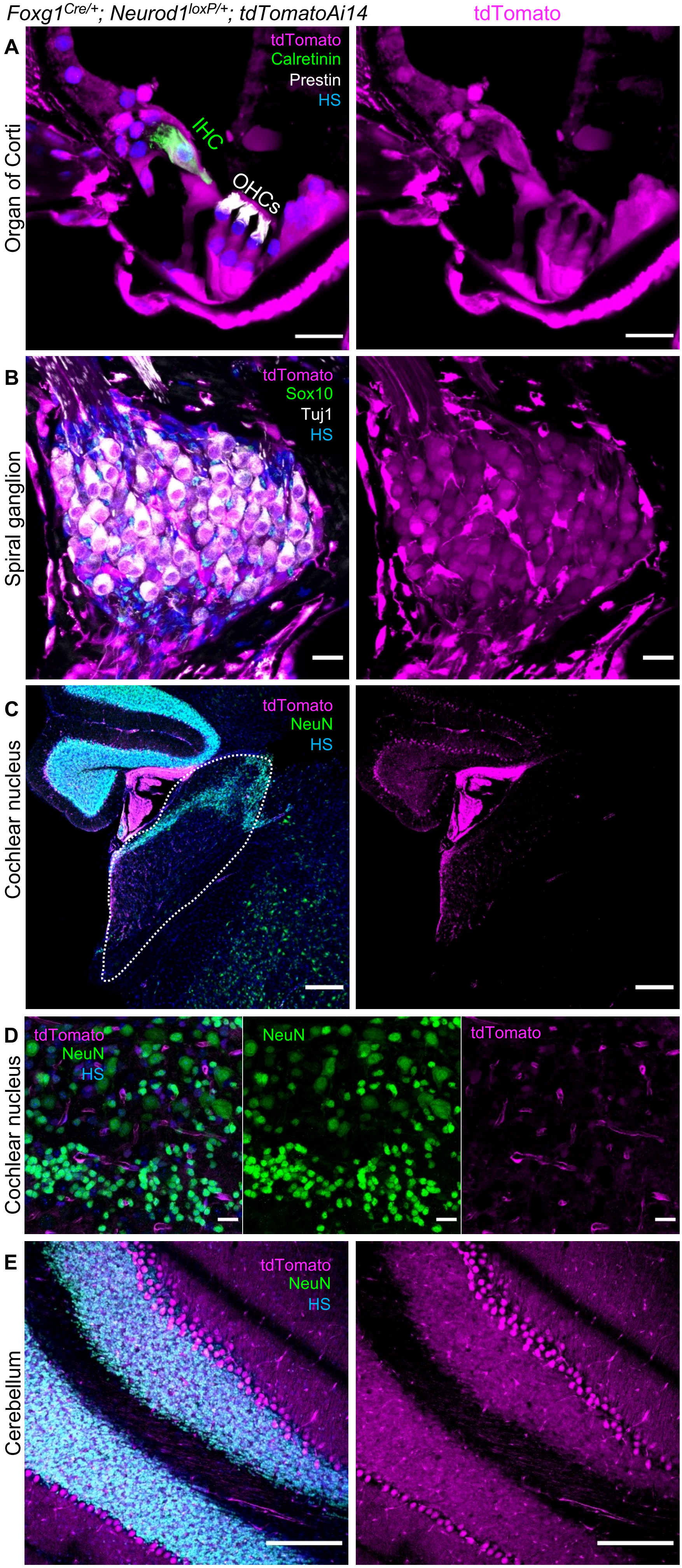

Supplementary Figure 1: The expression pattern of *Foxg1*^{Cre} shown by tdTomato reporter mice. Representative confocal images show tdTomato expression in *Foxg1*$^{Cre/+}$; *Neurod1*$^{loxP/+}$; *tdTomatoAi14* adult mice together with immunolabeled cell markers. (A) The section shows organ of Corti with inner hair cells (IHCs) labeled by anti-calretinin and outer hair cells (OHCs) labeled by anti-prestin co-expressing tdTomato. (B) The section of spiral ganglion shows anti-Sox10 labeled glial cells, and tdTomato^{+} expressing neurons with anti-Tuj1 labeled beta tubulin III in neurons and neurites. (C) The section of the cochlear nucleus immunostained with anti-NeuN, a marker of differentiated neurons. Dotted line indicates boundaries of the cochlear nucleus. (D) Higher magnification images demonstrate that neurons in the cochlear nucleus do not express tdTomato, confirming no expression of *Foxg1*^{Cre} in the cochlear nucleus. A merged image and images of individual colors of the separate channels are shown. (E) Higher magnification image shows tdTomato expression in the cerebellum. Note Purkinje cells expressing tdTomato^{+} and negative for NeuN. HS, Hoechst nuclear staining. Scale bars: 20 µm (A, B, D); 200 µm (C, E).

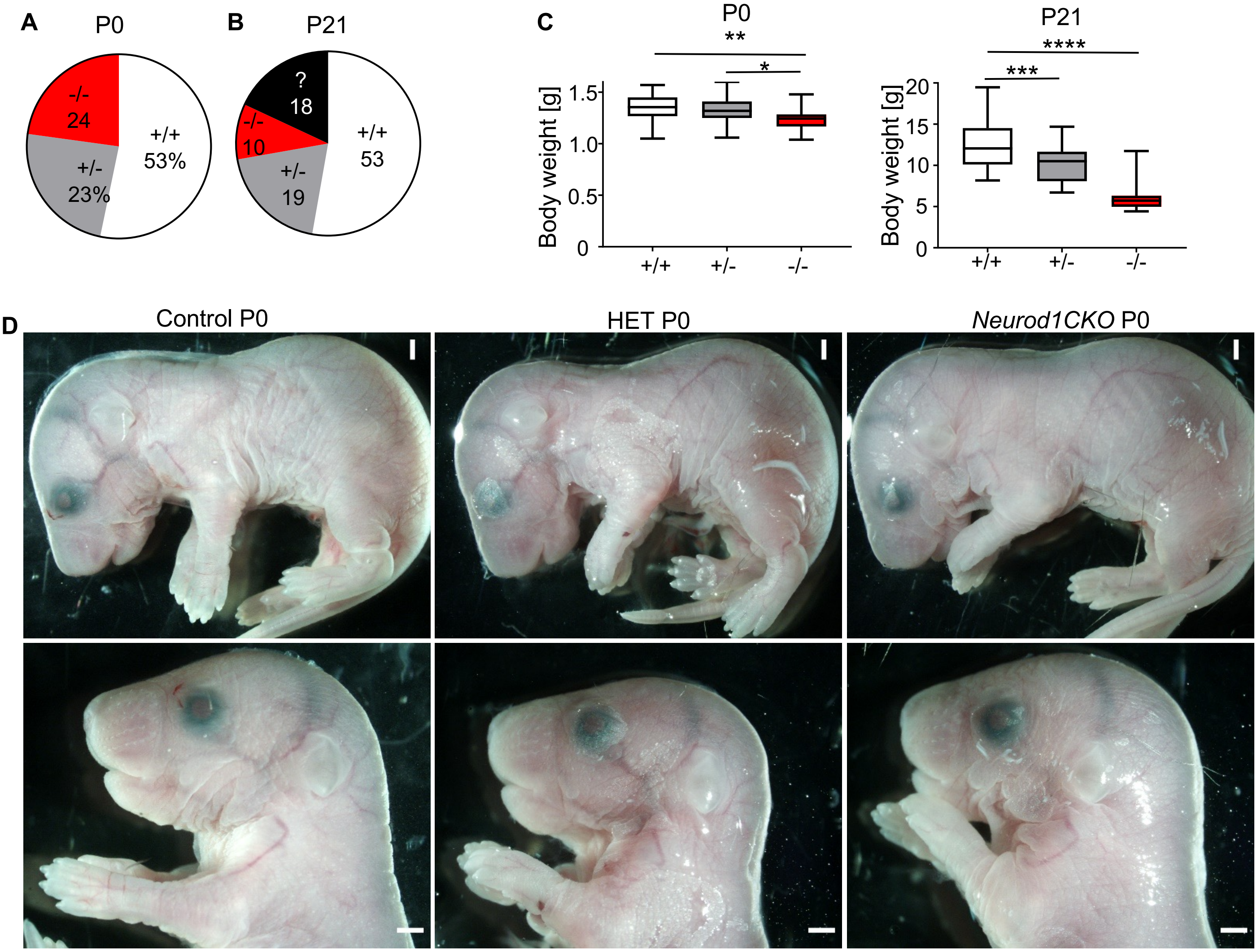

Supplementary Figure 2: Gross morphology. (**A**) Embryos were recovered at the Mendelian ratios of 53% controls without Cre expression (white, +/+), 23% heterozygous *Foxg1*$^{Cre/+}$, *Neurod1*$^{loxP/+}$ (HET; grey, +/-) and 24% homozygous *Neurod1CKO* mutants (*Foxg1*$^{Cre/+}$, *Neurod1*$^{loxP/loxP}$; red, -/-). (**B**) Postnatal survival of mutants was reduced compared to control mice, as analysed at 3 weeks after birth. (**C**) The body weight of *Neurod1* homozygous mutants was decreased compared to their littermate controls at the postnatal day 0 (P0), indicating abnormal embryonic development. The weight of both homozygous and heterozygous *Neurod1* mutants was significantly reduced at three weeks of age. Box plots indicate median (middle line), 25th, 75th percentile (box) and min to max (whiskers); one-way ANOVA, *P≤0.05, **P≤0.01, ***P≤0.001, ****P≤0.0001. (**D**) No noticeable gross external morphological abnormalities in a shape of the forehead or the snout, or in an eye formation are found in born pups. Scale bars: 1000 µm.

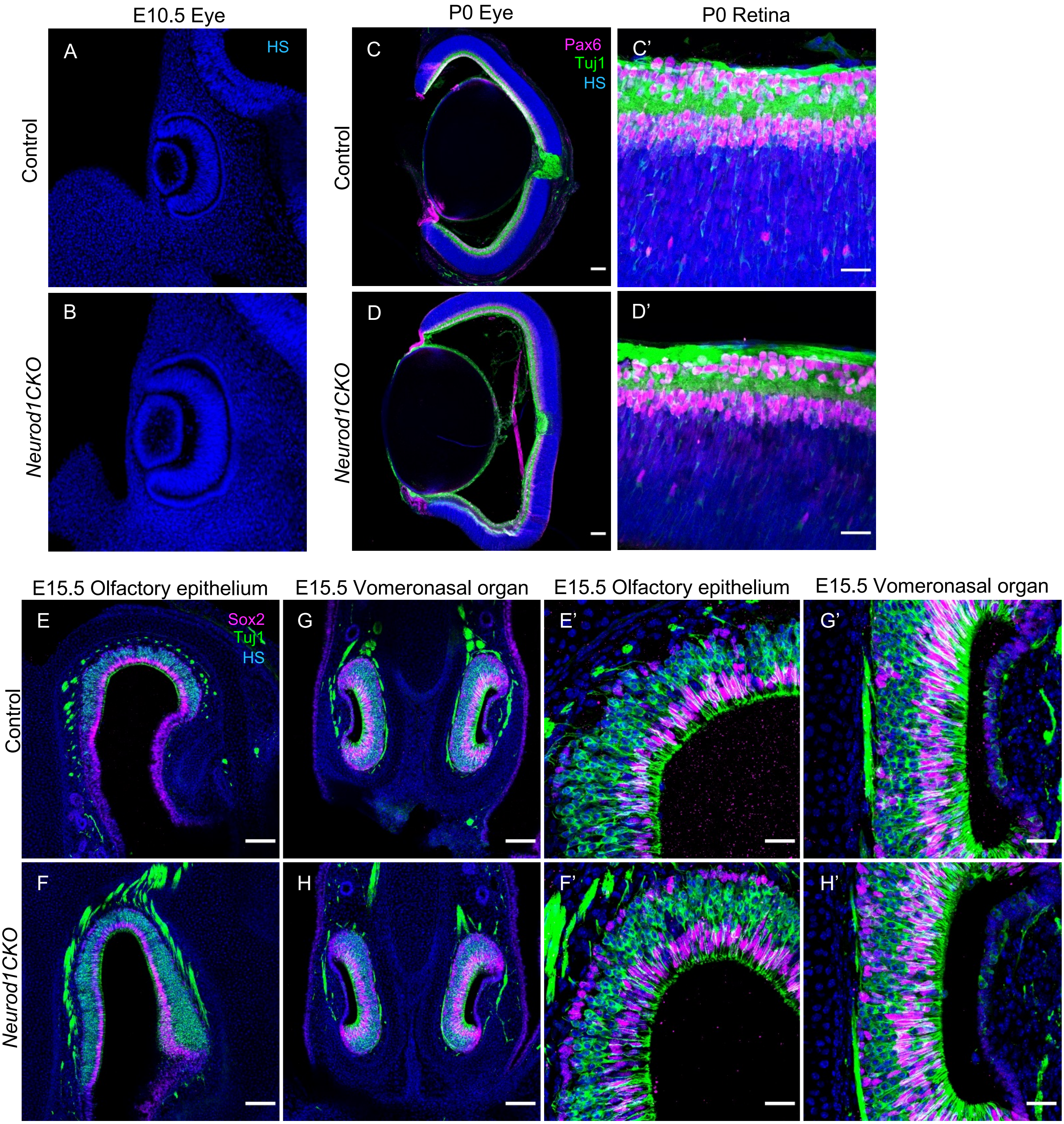

Supplementary Figure 3: Comparable eye and olfactory development between control and *Neurod1CKO.* Representative immunolabeled sections show comparable distribution of cells in the retina, olfactory and vomeronasal organ epithelia: Pax6, marker of retinal ganglion cells and amacrine cells; Tuj1, neuron specific tubulin; Sox2, Sus cells, globose basal cells and horizontal basal cells; HS, Hoechst nuclear staining. Scale bars: 100 μm (A-H); 25 μm (C'-H').

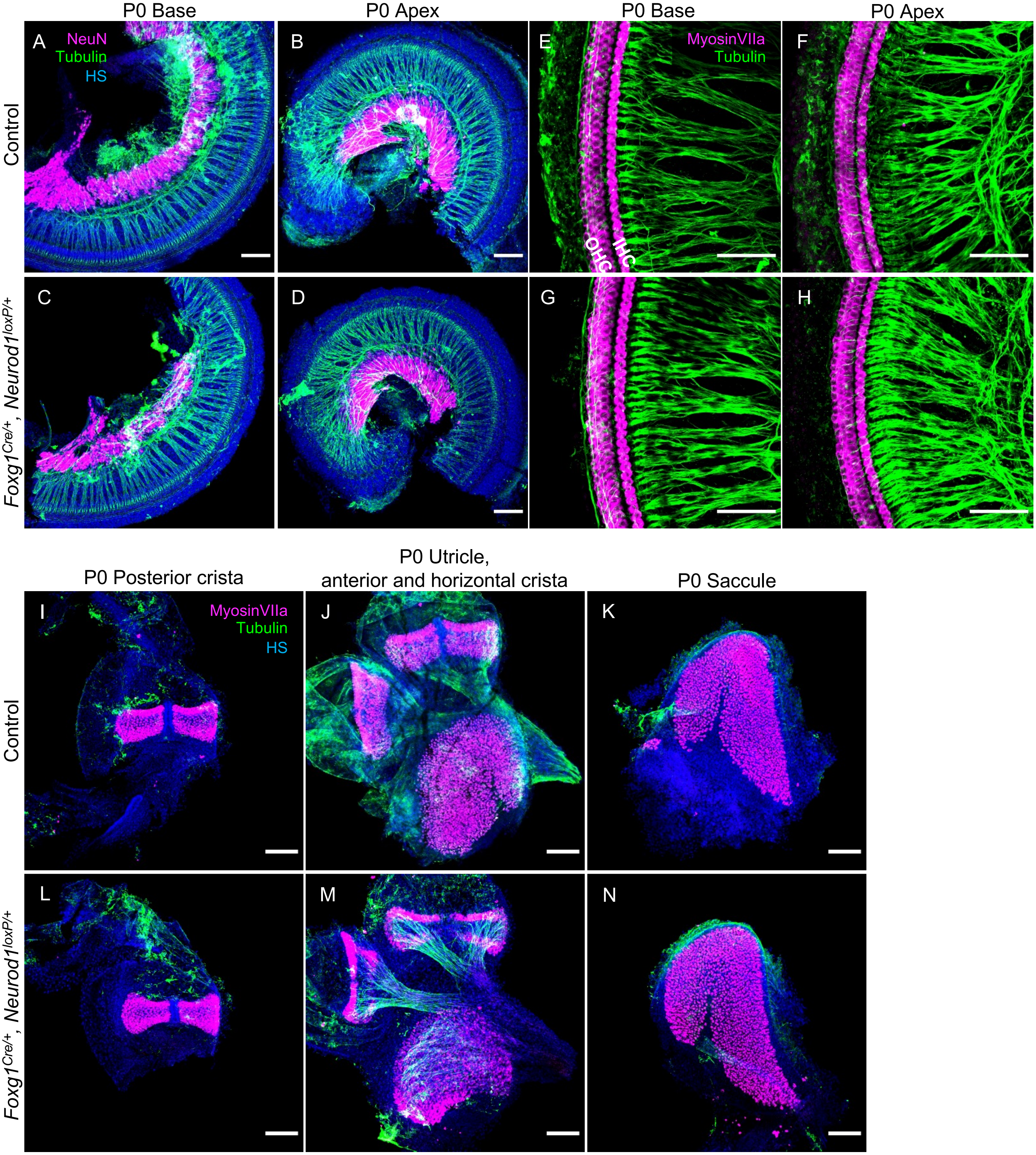

Supplementary Figure 4: Comparable inner ear phenotype between control and *Foxg1*$^{Cre/+}$, *Neurod1*$^{loxP/+}$ mice at P0. (A-D) The formation of spiral ganglion and radial fibers is comparable between the control and heterozygous *Neurod1* mutant cochlea (*Foxg1*$^{Cre/+}$;*Neurod1*$^{loxP/+}$). Neurons were labelled using anti-NeuN (marker of differentiated neurons) and neuronal fibers by anti-tubulin. **(E-H)** Representative confocal images from cochlear whole-mount preparations show in detail the sensory epithelium with hair cells labelled by anti-MyosinVIIa. One row of inner hair cells (IHC) and three rows of outer hair cells (OHC) are similarly innervated with afferents. **(I-N)** Representative images of whole-mount immunolabeling of the vestibular-end organs with anti-MyosinVIIa (a marker of hair cells) and anti-α-tubulin (nerve fibers) show comparable formation of sensory epithelia between the control and heterozygous *Foxg1*$^{Cre/+}$;*Neurod1*$^{loxP/+}$ mutant. Scale bars: 100 µm (A-D; I-N); 50 µm (E-H).

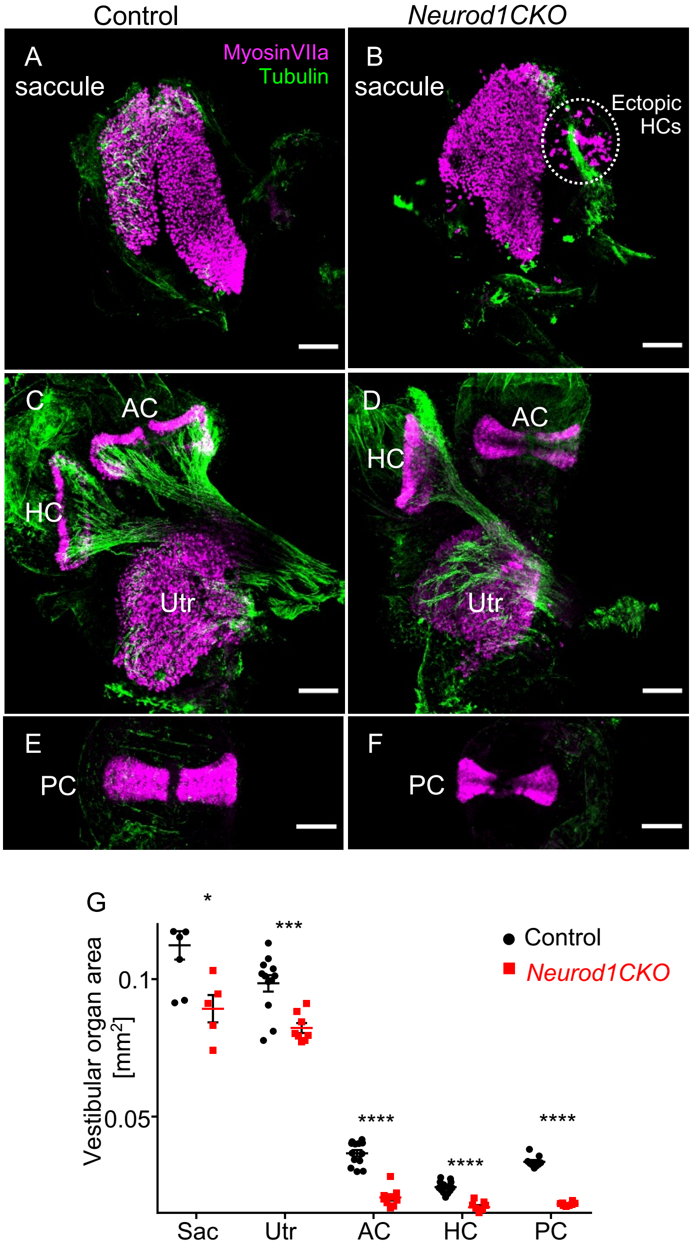

Supplementary Figure 5: Smaller sensory epithelia in the *Neurod1CKO* mutant inner ear. (A-F) The reduced size of sensory epithelia in *Neurod1CKO* compared to control littermates is shown in whole-mounts of the vestibular end-organs labelled by anti-MyosinVIIa (a marker of hair cells) and anti-α-tubulin (nerve fibers) at E18.5. Note ectopic hair cells in *Neurod1CKO* demarked by the white dashed line in (B). (**G**) Quantification of the area of vestibular end-organ sensory epithelia. Error bars represent mean ± SD; unpaired *t*-test. *P≤0.05, ***P≤0.001, ****P≤0.0001 (n ≥ 5/genotype). Scale bars: 100 µm. AC, anterior crista; HC, horizontal crista; PC, posterior crista; Utr, utricle.

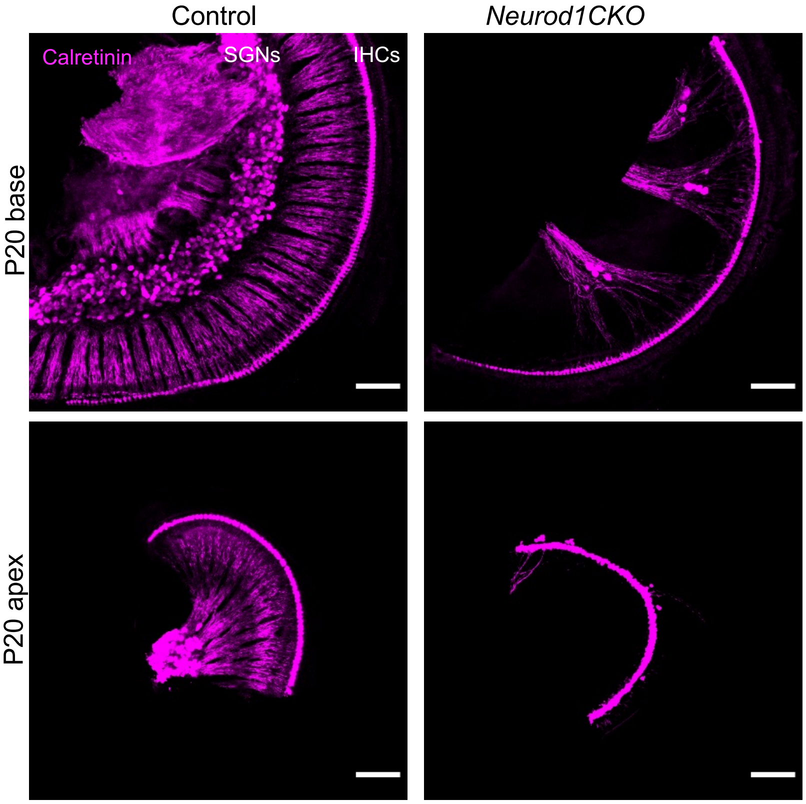

Supplementary Figure 6: Type Ia neurons and fibers found in the *Neurod1CKO* at P20. Number of neurons and innervation in the mutant cochlea are severely reduced compared to controls, as shown by whole-mount immunolabeling using anti-calretinin (a marker of IHCs and type Ia SGNs). IHCs, inner hair cells; SGNs, spiral ganglion neurons. Scale bars: 100 µm.

Supplementary Table 1: List of primary and secondary antibodies

Antibody name	Host	Producer	Catalog No	Dilution
Calbindin-D-28K	Mouse	Merck	C9848	1:250
Calretinin	Rabbit	Santa Cruz Biotechnology	sc-50453	1:100
Cleaved Caspase3 (Asp175)	Rabbit	Cell Signaling Technology	9661	1:100
Cre Recombinase	Rabbit	BioLegend	908001	1:500
Isl1 & Isl2	Mouse	Developmental Studies Hybridoma Bank	39.4D5	1:200
MyosinVIIa	Rabbit	Proteus BioSciences	25-6790	1:500
NeuN	Rabbit	Abcam	ab177487	1:500
Neurod1	Goat	Santa Cruz Biotechnology	sc-1084	1:100
Parvalbumin	Rabbit	Abcam	ab11427	1:2000
Pax6	Rabbit	BioLegend	901301	1:100
Phospho-Histone H3 (Ser10)	Rabbit	Merck	06-570	1:100
Prestin	Goat	Santa Cruz Biotechnology	sc-22692	1:50
Sox2	Goat	Santa Cruz Biotechnology	sc-17320	1:250
Sox10	Rabbit	Abcam	ab155279	1:250
Tubulin (acetylated α-tubulin)	Mouse	Merck	T6793	1:400
Tuj1 (Tubulin β3)	Mouse	BioLegend	801202	1:500
VGLUT1 (Vesicular Glutamate Transporter1)	Mouse	Merck	MAB5502	1:200
Alexa Fluor® 488 AffiniPure Donkey Anti-Mouse IgG (H+L)	Donkey	Jackson ImmunoResearch	715-545-150	1:500
Alexa Fluor® 594 AffiniPure Donkey Anti-Rabbit IgG (H+L)	Donkey	Jackson ImmunoResearch	711-585-152	1:500
Alexa Fluor® 647 AffiniPure Donkey Anti-Goat IgG (H+L)	Donkey	Jackson ImmunoResearch	705-605-147	1:500
Alexa Fluor® 488 AffiniPure Goat Anti-Mouse IgG (H+L)	Goat	Jackson ImmunoResearch	115-545-146	1:500
Alexa Fluor® 594 AffiniPure Goat Anti-Rabbit IgG (H+L)	Goat	Jackson ImmunoResearch	111-585-144	1:500
